# High-protein oral nutritional supplement use in patients with cancer reduces complications and length of hospital stay: a systematic review and meta-analysis

**DOI:** 10.3389/fnut.2025.1654637

**Published:** 2025-09-10

**Authors:** Marta Delsoglio, Rebecca Capener, Trevor R. Smith, Mhairi Donald, Gary P. Hubbard, Rebecca J. Stratton

**Affiliations:** ^1^Clinical Research, Nutricia Ltd., Trowbridge, United Kingdom; ^2^Department of Gastroenterology, University Hospitals Southampton NHS Foundation Trust, Southampton, United Kingdom; ^3^Sussex Cancer Centre, University Hospitals Sussex NHS Foundation Trust, Brighton, United Kingdom; ^4^Faculty of Medicine, University of Southampton, Southampton, United Kingdom; ^5^Division of Food, Nutrition & Dietetics University of Nottingham, Sutton Bonington, United Kingdom; ^6^Danone Global Research & Innovation Centre, Utrecht, Netherlands

**Keywords:** cancer, high-protein oral nutritional supplements, malnutrition, meta-analysis, nutrition support, oncology, ONS, systematic review

## Abstract

**Introduction:**

Oral nutritional supplements (ONS) have been reported to improve nutritional status, quality of life and clinical outcomes in many patient groups. This systematic review investigated the effects of high-protein ONS (HPONS), ≥20% energy from protein, on clinical outcomes in cancer patients.

**Methods:**

A systematic review (searches to January 2025) identified 32 publications reporting results from 29 randomised controlled trials (RCTs) (*n* = 2,279) of HPONS (mean daily intake 580 kcal, 34 g protein, ranging from 5 to 365 days) alongside dietary intake in patients with gastrointestinal (GI) (14RCTs), lung (4RCTs), head and neck (4RCTs), liver (2RCTs), breast (1RCT), and mixed (4RCTs) cancers across hospital and community undergoing surgery, chemotherapy, and/or radiotherapy. Studies reporting relevant outcomes (complications, length of hospital stay (LOS), hospital readmissions, and mortality) were pooled into a meta-analysis (Comprehensive Meta-Analysis software v4).

**Results:**

Meta-analysis showed a significant overall reduction (101 fewer per 1,000 patients) in complications (15RCTs, *n* = 1,230), including infectious, non-infectious and post-operative complications, and radiotherapy-related toxicities in patients using HPONS undergoing surgery and/or chemo/chemo-radiotherapy (OR: 0.62, 95% CI: 0.48-0.81; *p* = 0.0005; *I*^2^ = 0%) vs. control. The number needed to treat for preventing one additional complication with HPONS was 12 (95% CI: 9-29). A sub-group of studies (9RCTs) with HPONS enriched with omega-3 fatty acids also showed a positive effect on complications vs. control (OR: 0.69, 95% CI: 0.51-0.93; *p* = 0.02; *I*^2^ = 16%). A reduction in LOS was observed (8RCTs, *n* = 865) with HPONS (−0.26 days, 95% CI: −0.49 to −0.03; *p* = 0.02, *I*^2^ = 60%), while no significant difference was detected in hospital readmissions (5RCTs, *n* = 479) and mortality (7RCTs, *n* = 694).

**Conclusion:**

This systematic review and meta-analysis provide evidence that the use of HPONS (including those enriched with omega-3 fatty acids) alongside dietary intake is associated with a significant reduction in complications and LOS in cancer patients. The review found no significant effects on hospital readmissions or mortality. Given the heterogeneity of the patient population, further investigation is needed to comprehensively evaluate the effect of nutritional support on patient outcomes according to specific cancer and treatment types in various clinical settings.

## 1 Introduction

Cancer, a leading cause of death worldwide, is a complex disease that poses significant challenges to healthcare systems and patients alike. One important issue faced by patients with cancer is malnutrition, a condition that can exacerbate the disease's severity, hinder treatment effectiveness, and negatively impact patients' clinical outcomes (1–3).

Malnutrition in patients with cancer is a multifaceted problem, accounting for 20% of cancer deaths (4). Estimates suggest that between 5 and 85% of patients with cancer are malnourished (5), with the prevalence varying hugely according to cancer type and stage of disease, being greatest in cancers of the pancreas, stomach, oesophagus, head and neck, and lung (6–8). The accompanying loss of muscle mass and strength (sarcopenia) (9) is common (estimated overall prevalence of 35%, >50% in oesophageal, urothelial, cholangiocarcinoma, prostate, and thyroid cancer patients) (10), leading to functional impairments and poorer clinical outcomes in malnourished patients (11–13). The metabolic demands of cancer, coupled with the side effects of surgery and treatments such as chemotherapy and radiotherapy, often lead to decreased appetite, altered taste perception, and other factors that contribute to inadequate dietary intakes (14, 15), leading to malnutrition and, for some patients, cancer cachexia (16, 17). Consequently, a critical part of the management of cancer patients with or at risk of malnutrition is to improve nutritional intakes, where clinically indicated, including the use of nutritional support that is tailored to the specific cancer population, treatment modality and disease- and treatment-related side effects (18–20). Recent guidelines by the European Society for Clinical Nutrition and Metabolism (ESPEN) advocate increased attention to nutritional support in all patients with cancer and make recommendations according to treatment modality (19). The European Society for Medical Oncology (ESMO) clinical practice guidelines recommend nutritional support for patients with cancer cachexia. This includes dietary counselling, advice on selecting high-energy, high-protein foods, enriching meals, and using oral nutritional supplements (ONS) to increase energy intake and promote weight gain (21).

Multi-nutrient ONS, which are designed to provide a concentrated source of energy and nutrients, are often recommended alongside dietary advice to optimise nutritional intakes and reduce malnutrition risk (19, 21–23). ONS have been reported to improve nutritional status, quality of life and clinical outcomes in various cancer patients (23–28). In particular, the use of ONS has been shown to reduce the post-operative inflammatory response, enhance immune function, and improve the nutritional status of gastrointestinal (GI) cancer patients (29–31), as well as decrease the risk of post-operative complications and hospital length of stay (LOS) (32). Similarly, oral nutritional supplementation has indicated promising results on the post-operative course of patients undergoing hepatic resection for malignancy (33), as well as positively influencing mortality, treatment tolerance, quality of life, functional status, and adverse events in head-and-neck cancer patients (34).

However, for the dietary management of patients with cancer, use of ONS that are high in protein (≥20% of energy from protein) may be warranted to better improve inadequate protein intake associated with anorexia, especially considering protein requirements are often elevated due to the metabolic derangements from both the tumour and cancer treatment (16, 35). A protein intake above normal may be desirable to counteract increased protein losses, sarcopenia, to encourage repair of damaged tissues, and to support immune function (36). Protein catabolism from muscle mass and cancer-related malnutrition during cancer treatment has been associated with reduced quality of life (37). Consuming at least the minimum amounts of protein and energy recommended has been shown to help prevent weight loss and improve nutritional status (38).

ESPEN and the American Society for Parenteral and Enteral Nutrition (ASPEN) have established evidence-based guidelines for nutrition management and specifically for weight loss prevention in cancer patients, both advising consumption of 1 g/kg/day of protein and, if possible, up to 1.5 g/kg/day (19). Indeed, protein intakes below 1.2 g/kg/day, even when within the recommendations, have been associated with muscle wasting during cancer treatment, with only intakes above 1.4 g/kg/day being associated with muscle maintenance (39). According to a recent literature review, the dose of amino acids capable of supporting a positive protein balance in cancer patients might be closer to 2 g/kg/day (40), and so it is likely that high-protein nutritional support will be needed in addition to the diet to help patients meet such requirements. Clinical, nutritional, and functional benefits from high-protein oral nutritional supplement (HPONS) use have been demonstrated in a range of patient groups and health settings, including reduced complications, reduced readmissions to hospital, improved grip strength, increased intake of protein and energy with little reduction in normal food intake, and improvements in weight (41, 42). In hospitalised cancer patients, HPONS have been shown to improve functional outcomes and quality of life, as well as reduce mortality (43). Some HPONS are also enriched with anti-inflammatory n-3 long-chain polyunsaturated fatty acids (n-3 PUFAs). The administration of eicosapentaenoic acid (EPA), a long-chain PUFA of the omega-3 (n-3) family, has been highlighted as a potentially beneficial approach to further improve the management of patients with cancer (44–47). There is evidence that the consumption of n-3 PUFA-enriched ONS exert beneficial effects in patients undergoing chemo (radio) therapy, including an increase in body weight, BMI, and a significant reduction in plasma levels of C-reactive protein (CRP), tumour necrosis factor-α (TNF-α), interleukin 6 (IL-6), and the incidence of adverse events (48).

However, overall, studies are heterogeneous, and questions remain regarding the use of HPONS in cancer patients, particularly their effects on clinical outcomes. Although previous systematic reviews investigated the effect of high-protein interventions, including foods, on a range of nutritional outcomes (49), to inform clinical decisions on the use of HPONS in cancer patients, it is important to specifically review the up-to-date evidence for their effects on clinical outcomes. Therefore, this systematic review aimed to critically review and assess the impact of HPONS, including those enriched with omega-3 fatty acids, on clinical outcomes, including complications, length of hospital stay, readmissions to hospital, and mortality in patients with cancer.

## 2 Subjects and methods

The review was planned, conducted and reported following published guidelines (50, 51).

### 2.1 Eligibility criteria

Studies were eligible for inclusion if they matched the pre-determined inclusion and exclusion criteria (for full details, see Table 1). In brief, clinical studies were restricted to randomised controlled trials (RCTs) undertaken in all cancer patients, published in English as a full manuscript (abstracts and conference proceedings were excluded). Participants included adults (mean age ≥18 years) of any nutritional status (well-nourished, malnourished, or mixed), randomised to receive the intervention in any setting. In terms of intervention, all studies using multi-nutrient (at least two macronutrients and one micronutrient), high-protein (≥20% energy from protein) (52) ONS (including those simultaneously using dietary advice and/or standard diet and/or ONS not high in protein) were eligible to be included. All types of ONS were permitted (powder and ready-made formulas). Studies were excluded if they assessed enteral tube feeding, dietary counselling only, parenteral nutrition, or those where ONS was used as a meal replacement to promote weight loss, or when ONS was used in combination with another intervention (e.g., exercise) where the effect of the HPONS alone could not be determined.

**Table 1:** Eligibility criteria for the systematic review.

**Criteria**	**Include**	**Exclude**
Population	• Adults 18 years and over • Any nutritional status (well-nourished, malnourished, or mixed) • Based in any setting (hospital, community) • Any sample size • Cancer patients (all cancer types at any stage of treatment)	• Animal studies • Developing world • Pregnancy and lactation • Sports studies • Kinetic studies • Healthy adults
Intervention/comparator	• Multi-nutrient high protein (≥20% of total energy from protein) (52) oral nutrition supplements of any consistency (including those simultaneously using or comparing with dietary counselling and/or standard diet and/or standard nutritional supplement not high in protein) • ONS must contain at least two macronutrients and one micronutrient • Nutritional support can be nutritionally complete or incomplete and provide some or all of the entire daily requirement • Any duration of intervention	• Dietary counselling only • Enteral tube feeding only • Parenteral nutrition only • Nutrition support with < 2 macronutrients, no micronutrients, < 20% energy from protein • Amino acid-based formula • Combination studies if can't determine the benefit of nutrition support only, e.g., where nutrition support is part of an intervention package • ONS used for weight reducing diet
Outcomes	• Clinical (infections, post-operative complications, chemo/radiotherapy related toxicities) • Healthcare use (length of stay, hospital readmissions) • Mortality • Compliance with supplementation • Dietary intake (energy and protein intake)	
Study design/publication type	• Randomised controlled trials (RCTs) published in full text	• All non-RCT study designs • Conference abstracts (must be full papers)
Language of publication	• English	• Non-English language publications
Countries	• No restriction	• None

### 2.2 Search strategy

A systematic literature review was performed in accordance with PRISMA guidelines (51) to identify RCTs that assessed the effect of HPONS on nutritional, clinical and functional outcomes. The search strategy was developed in Ovid MEDLINE using relevant free text and MeSH terms and was then modified for searches in EMBASE and the Cochrane Library. Searches of MEDLINE (Ovid MEDLINE Epub Ahead of Print, In-Process & Other Non-Indexed Citations, Ovid MEDLINE, Daily and Ovid MEDLINE), Embase, and the Cochrane Library (EBM Reviews: Cochrane Central Register of Controlled Trials, Cochrane Database of Systematic Reviews, Database of Abstracts of Reviews of Effects, Health Technology Assessments) were performed up to 9th January 2025.

Duplicate records were identified and removed prior to title and abstract screening. Following a study screening hierarchy for exclusion, all titles and abstracts identified through the literature searches were screened by three reviewers to assess whether they met the eligibility criteria. Once title and abstract screening were completed, the reviewers reconciled any existing discrepancies between their selections of studies.

At the full-text screening stage, where multiple publications reported the same RCT, all relevant reports were retained to ensure comprehensive data capture. These publications were carefully reviewed and linked to their corresponding parent study, allowing extraction of complementary information while avoiding duplication in the synthesis. The same three reviewers independently screened full-text articles for all studies identified as included at the title and abstract screening phase. When a consensus could not be reached between the three reviewers during reconciliation processes, a senior reviewer provided arbitration. The reviewers discussed any differences of opinion before deciding on the final list of included/excluded articles.

### 2.3 Quality assessment

Risk of bias in included studies was judged by two independent reviewers using the revised Cochrane tool for assessing risk of bias in randomised trials (RoB2 tool) (53); all disputes were resolved by discussion and consensus. The likelihood of bias was judged across five domains: (1) bias arising from the randomisation process, (2) bias due to deviations from intended interventions, (3) bias due to missing outcome data, (4) bias in measurement of the outcome, and (5) bias in selection of the reported result. The judgements within each domain lead to an overall risk of biased judgement for the outcome being assessed. Studies were judged to be either at low overall risk when no bias was detected across all domains, high overall risk of bias in case there was a high risk of bias in at least one domain or having some concerns for at least one domain due to insufficient information provided.

Grades of Recommendation, Assessment, Development, and Evaluation (GRADE) criteria were used to assess the overall strength of evidence for the pooled outcomes (54). Data from included RCTs, which were quality assessed using RoB2, were initially rated as high. Evidence was further downgraded for one or two levels in the presence of (1) risk of bias (if >33.3% of the weight in a meta-analysis came from studies at moderate and high risk of bias or more than 33% of the weight came from high risk of bias, respectively), (2) indirectness (if >33.3% of the weight in a meta-analysis came from partially indirect or indirect studies, respectively), (3) inconsistency (if the *I*^2^ was >33.3% and 66.7%, respectively), (4) imprecision [if the 95% confidence interval for the effect size crossed one or both lines of the minimal clinically important difference (MID) threshold], and (5) publication bias (if the funnel plot showed suspicion or convincing evidence of publication bias).

Each RCT was also rated into one of three groups for directness, if there were concerns about how directly the population, intervention, comparator, and/or outcomes (PICO) in the study could address the specified review question: direct (if no important deviations from the eligibility in PICO); partially indirect (if important deviations in one of the PICO criteria); and indirect (if important deviations from the protocol in at least two of the PICO criteria). To identify published MID thresholds relevant to this review, the Core Outcome Measures in Effectiveness Trials (COMET) database (https://www.comet-initiative.org) was searched (9th January 2025). However, as MIDs were not available, a default clinical decision threshold for dichotomous outcomes of 0.8–1.25 was used, in line with the National Institute for Health and Care Excellence methods (55). To assess and graphically present publication bias and its possible effect on the performed meta-analyses, funnel plots were used (not shown). As the statistical power of trials is determined by factors other than sample size, such as the number of participants experiencing the event, the standard error of the intervention effect estimate (*Y*-axis) was plotted against the log of the odds ratio (*X*-axis) (56). Funnel plots were generated when ten or more studies were combined in a meta-analysis using Review Manager version 5.4.1 (57). Based on all five GRADE-criteria, the overall quality of evidence was rated as either: high, we are very confident that the true effect lies close to that of the estimate of the effect; moderate, we are moderately confident in the effect estimate; low, our confidence in the effect estimate is limited; or very low, we have very little confidence in the effect estimate. The GRADE analysis was performed using the GRADEpro software (58).

### 2.4 Data extraction and synthesis

A pre-determined data extraction table was designed to capture all key study characteristics, including patient population age and characteristics, intervention HPONS type, duration, dose prescribed, and comparator arm. If the data on continuous outcomes were reported as medians and range or interquartile range, the mean and standard deviation were estimated according to Luo and Wan's methods (59, 60).

Studies were classified according to the setting in which the intervention was consumed: “community,” the intervention was administered in the community only; “hospital,” the intervention was administered in the hospital only, “community-hospital” the intervention was administered in the community before hospital admission and continued during hospital admission, “community-hospital-community” the intervention commenced in the community before hospital admission, continued during admission and after discharge, “hospital-community” the intervention commenced in hospital and then continued in the community.

Comparator arm was categorised into standard care (normal diet, routine care, *ad libitum* diet, and hospital diet), dietary advice/counselling (DC), non-nutritious placebo, isocaloric diet, and standard ONS (< 20% of total energy from protein).

Outcome measures sought included clinical and healthcare use: clinical complications (infections, post-operative complications, chemo/radiotherapy related toxicities, etc.), healthcare use (length of stay, hospital re-admissions), mortality, compliance with HPONS supplementation, and dietary intake (energy and protein intake).

Complications were defined by each study and included infections (respiratory infections, gastrointestinal infections, cardiac infections, and renal infections), general post-operative complications, and radio/chemotherapy-related toxicities. For the purpose of this systematic review, gastrointestinal side effects and non-specific symptoms were not included as complication data.

Compliance was defined as the percentage of the HPONS actually consumed by the patients relative to the amount prescribed.

Following the extraction of data from eligible studies, meta-analysis was conducted where appropriate and feasible, for comparable trials with numerically consistent outcome measures (trials reporting the same outcomes in the same way).

Any discrepancies observed between the data extracted by the two analysts were adjudicated by a third reviewer. All studies with the relevant outcome were eligible for synthesis and are described in the results.

### 2.5 Meta-analysis and statistics

Data on complications, length of hospital stay (LOS), readmissions to hospital, and mortality were extracted, and a meta-analysis was performed. Exploratory sub-group meta-analyses were undertaken to investigate the impact of omega-3-enriched HPONS. Comprehensive Meta-Analysis (version 4, Biostat) (61) was used to undertake planned meta-analysis on the incidence of complications and mortality. Heterogeneity between comparable trials was explored using the *I*^2^ test (62, 63) using more than 50% as the cut-off for heterogeneity. A fixed-effects model was used when *I*^2^ was below 50%, and a random-effects model was used when *I*^2^ was above 50%. Categorical data are presented as odds ratios (ORs) and 95% confidence intervals (CIs), continuous data are presented as standardised mean differences (SMDs) with overall significance assumed at *p* < 0.05. Forest plots are used to present the data. Sensitivity analyses were performed to explore the influence of poor study quality and study size. Outcome data that could not be included in the meta-analysis are described in the text. The number needed to treat (NNT) was calculated to quantify the clinical impact of HPONS on reducing complications. NNT was calculated as the inverse of the absolute risk reduction (ARR), defined as the difference between the control event rate (CER) and experimental event rate (EER) (64). Event rates were derived from pooled data across included studies.

## 3 Results

### 3.1 Overall search findings

**Figure 1:**
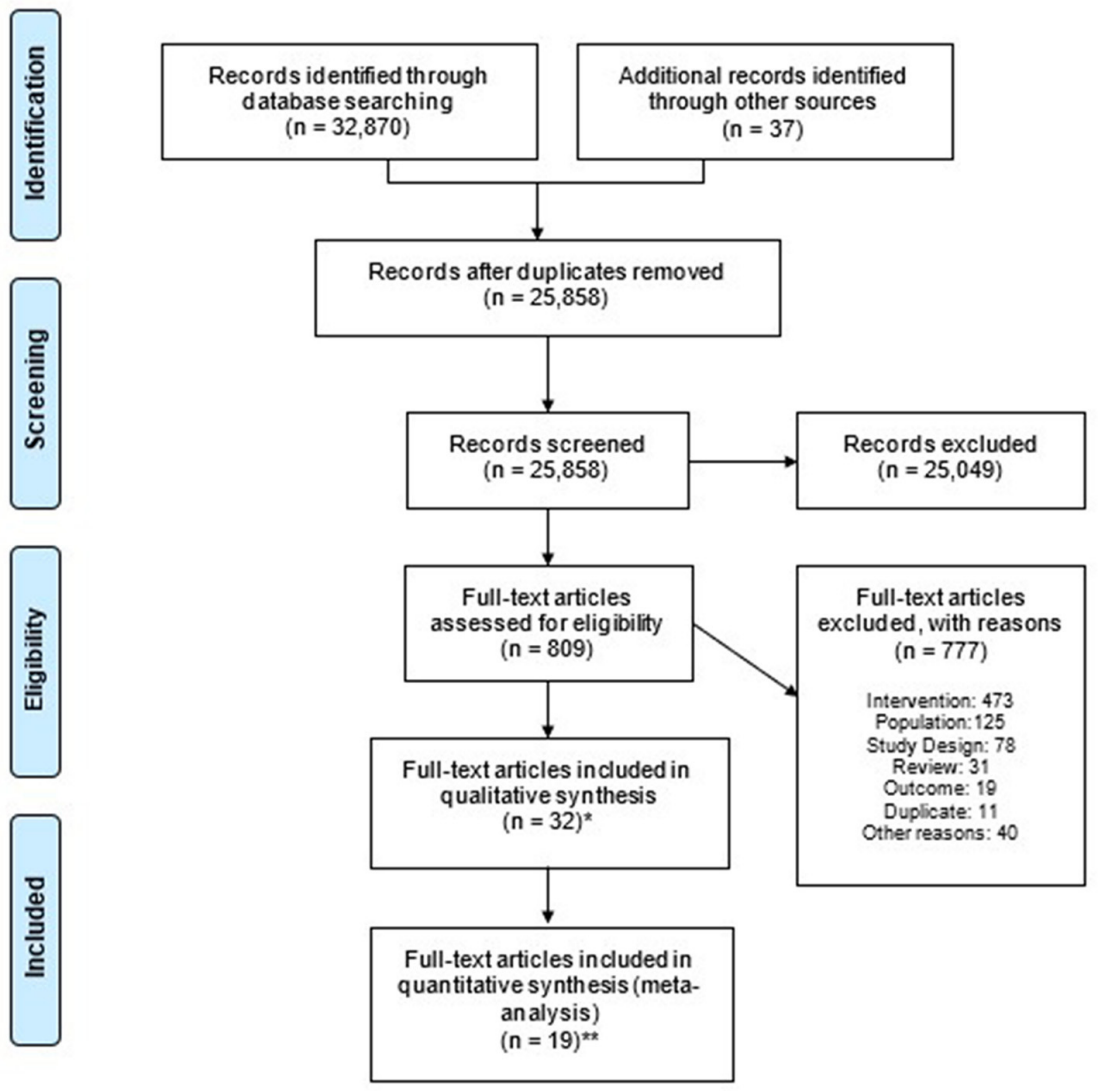
Flowchart summarising the systematic review (PRISMA). PRISMA 2020 flow diagram illustrating the study selection process. At the title and abstract screening stage, records were excluded without recording individual reasons, in accordance with PRISMA guidelines. Duplicate publications of the same RCT were identified and retained. A total of 32 publications (of 29 studies) were identified as eligible for inclusion in the review, and 19 publications (of 19 studies) reported at least one relevant clinical outcome (complications, LOS, hospital readmissions, and mortality) for inclusion in meta-analysis. ^*^n of publications of *n* = 29 studies. ^**^Number of publications included in quantitative synthesis per relevant outcomes, *n* = 19 studies.

A total of 32,907 publications were identified. After removing 7,049 duplicates, 25,858 publications were deemed eligible for the first review. On the basis of title and abstract, 25,049 were excluded at screening (Figure 1). The remaining 809 had the full text assessed, and 777 publications were excluded for the following reasons: intervention (e.g., not high protein, not ONS) (*n* = 473), study population (e.g., healthy, non-cancer patients) (*n* = 125), study design (e.g., not an RCT) (*n* = 78), review article (*n* = 31), outcome (e.g., no relevant nutritional, functional, clinical outcomes) (*n* = 19), study duplicate (*n* = 11), and other reasons (*n* = 40). In total, 32 publications (of *n* = 29 studies) were identified as eligible for inclusion in the review (65–96), and 19 publications (of *n* = 19 studies) reported relevant outcomes (complications, LOS, hospital readmissions, and mortality) for inclusion in meta-analysis (65, 68, 73–75, 77–82, 84, 88, 89, 91, 92, 94–96). For the two studies (81, 94) that used a standard ONS as a comparator, a separate meta-analysis on complications was conducted, and they were excluded from the main meta-analyses on mortality (81) and hospital readmission (94). One study reported relevant outcomes (complications and LOS) and was included in the meta-analysis despite the fact that part of the control group received standard ONS alongside standard care, as it was not possible to determine how many patients took the ONS (73).

### 3.2 Number of publications vs. number of studies

The thirty-two publications reporting results from twenty-nine studies (*n* = 2,279) eligible for inclusion in the systematic review are shown in Table 2. A complete list of references, along with relevant outcomes, is provided in Supplementary material. Two publications reported long-term outcomes of original studies; Aoyama et al. (67) reported long-term survival and surgical morbidity at 3 and 5 years for the cohort in Ida et al. (78), and Ravasco et al. (88) reported survival at 6.5 years for the cohort in Ravasco et al. (87). Van der Meij et al. (94) investigated different outcomes (complications, readmissions to hospital) for the same study as van der Meij et al. (93), which reported on compliance.

**Table 2:** Characteristics of publications in the systematic review (*n* = 32) arranged according to the first author's surname.

**Study**	**Population**	**Nutritional Status**	**Cancer treatment**	**Sample size**		**Age (y)**	**Setting**	**HPONS characteristics**	**Prescribed**	**Duration of intervention**	**Follow up**	**Control group**
				**R**	**A**							
Abouegylah et al. (65) (Egypt, Germany)	Head and neck cancer	Not malnourished (excluded pre-existing malnutrition & BMI >30 kg/m^2^)	Chemoradiotherapy	I: 15 C: 15	I: 15 C: 15	I:66 C:55	Community	2.15 kcal/ml 20 En%P cow's milk protein (Medidrink Onco)	440 kcal 22 g P	42-49 days	Not reported	Normal diet (SC)
Akita et al. (66) (Japan)	Pancreatic cancer	Not reported	Chemoradiotherapy	I: 31 C: 31	I: 31 C: 31	I: 67.8 C: 66.4	Community	1.27 kcal/ml 20 En%P cow's milk protein 0.75 g omega-3 (0.45 g EPA)/100 ml (Prosure, Abbott)	560 kcal 29.3 g P	35 days	35 days	Normal diet (SC)
Aoyama et al. (67)^*^ (Japan)	Gastric cancer	Not reported	Cancer surgery	I: 63 C: 63	I: 63 C: 60	I: 65.1^§^ C: 65.6^§^	Hospital	1.27 kcal/ml 20 En%P cow's milk protein 0.75 g omega-3 (0.45 g EPA)/100 ml (Prosure, Abbott)	600 kcal 29.3 g P	28 days	1 and 3 months	Normal diet (SC)
Braga et al. (68) (Italy)	Colorectal cancer	Mixed	Cancer surgery	I: 50 C: 50	I: 50 C: 50	I: 63 C: 62.2	Community-Hospital	1.2 kcal/ml 22 En%P whey protein 0.33 g omega-3/100 ml (Oral Impact, Novartis)	1,236 kcal 67.2g P	5 days (pre-op group)	30 days	Ad Lib diet (SC)
Dingemans et al. (69) (Netherlands, Lithuania, Belgium, Norway)	Colorectal and lung cancer	Not malnourished (excluded >10% weight loss in past 6 months and BMI < 20 kg/m^2^)	Chemotherapy, chemoradiotherapy or immunotherapy	I: 28 C: 14	I: 26 C: 11	I: 66.1 C: 70.1	Community	2.4 kcal/ml 24 En%P cow's milk protein (Fortimel Compact Protein, Nutricia)	600 kcal 36 g P	84 days	84 days	Routine care (SC)
Dou et al. (70) (China)	Nasopharyngeal carcinoma	Mixed	Chemoradiotherapy	I: 26 C:26	I:23 C: 19	I: 48^§^ C: 47^§^	Community	3.9 kcal/ml 48 En%P (Healing Element, Methuselah Medical Technology)	493 kcal 60 g P	42 days	6 weeks	Dietary counselling
Faber et al. (71) (Netherlands)	B) Oesophageal cancer or adenocarcinoma of the gastro-oesophageal junction	Mixed	Chemo/radiotherapy	I: 13 C: 16	I:11 C: 16	I: 61.1 C: 61.6	Community	1.63 kcal/ml 24 En%P 0.61 g EPA/100 ml cow's milk protein (Forticare, Nutricia)	652 kcal 39.6 g P	28 days	4 weeks	Non-nutritious placebo + Dietary counselling
	C) Oesophageal cancer or adenocarcinoma of the gastro-oesophageal junction		Chemo/radiotherapy	I: 18 C:17	I:13 C: 7							Standard ONS + Dietary counselling
Faccio et al. (72) (Brazil)	Colorectal, breast, lung, upper digestive tract, ovarian, and other cancer	Not reported	Chemo/chemoradiotherapy	I: 44 C:45	I: 43 C: 42	I: 59.2^§^ C: 58.4^§^	Community	1.02 kcal/ml 25 En%P 0.27 g omega-3/100 ml whey protein (Immax, Prodiet)	630 kcal 38.2 g P	28 days	4 weeks	Dietary counselling
Gade et al. (73) (Denmark)	Pancreatic cancer	Mixed	Cancer surgery	I: 25 C: 21	I: 19 C: 16	I: 68^§^ C: 69^§^	Community–Hospital	1.2 kcal/ml, 22 En%P 0.33 g omega-3/100 ml whey protein (Oral Impact, Nestle)	878 kcal 47.7 g P	7 days	30 days	Dietary counselling with individual advice for standard ONS if at risk of malnutrition
Gianotti et al. (74) (Italy)	Gastrointestinal cancer	Excluded those with weight loss 10% in past 6 months	Cancer surgery	I: 102 C:102	I: 102 C: 102	I: 62.3 C: 63.4	Community–Hospital	1.0 kcal/ml, 23 En%P whey protein 0.33 g omega-3/100 ml (Oral Impact Powder, Nestle)	1,236 kcal 72 g P	5 days (pre-op group)	3 months	Normal diet (SC)
Hanai et al. (75) (Japan)	Head and neck cancer	Nutritional risk (≥5% weight loss in past 6 months in inclusion)	Cancer surgery	I: 14 C: 14	I: 13 C: 14	I: 61.5 C: 66.1	Community–hospital–community	1.25 kcal/ml, 21.3 En%P cow's milk protein 0.75 g omega-3 (0.45 g EPA)/100 ml (Prosure, Abbott)	600 kcal 32 g P	28 days	28 days	Routine care (SC)
Hatao et al. (76) (Japan)	Gastric cancer	Mixed	Cancer surgery	I: 92 C:65	I: 64 C: 49	I: 65.5^¶^ C: 63.9^¶^	Hospital–community	1.0 kcal/ml 20 En%P (ANOM, Otsuka Japan)	400 kcal 20 g P	84 days	12 weeks	Normal diet (SC)
Ibrahim et al. (77) (Egypt)	Liver cancer	Malnourished	Cancer surgery	I: 21 C: 22	I: 20 C: 20	I: 54.5 C: 57.3	Hospital	2.0 kcal/ml, 20 En%P (ONS name not reported)	35–40 kcal/kg 1.2–1.5 g/kg P	7 days	7 days	Hospital diet (SC)
Ida et al. (78) (Japan)	Gastric cancer	Not reported	Cancer surgery	I: 63 C: 63	I: 63 C: 60	I: 65.1^§^ C: 65.6^§^	Hospital	1.27 kcal/ml 20 En%P cow's milk protein 0.75 g omega-3 (0.45 g EPA)/100 ml (Prosure, Abbott)	600 kcal 29.3 g P	28 days	1 and 3 months	Normal diet (SC)
Kabata et al. (79) (Poland)	Gastric, colorectal, ovaries, pancreatic, appendix, and liver cancer	Not malnourished (according to their inclusion criteria)	Cancer surgery	I: 54 C:48	I: 54 C: 48	I: 60^§^ C: 67^§^	Community	1.5 kcal/ml, 26.7 En%P cow's milk protein, soy and pea protein isolate (Nutridrink Protein, Nutricia)	600 kcal 40 g P	14 days	30 days	Normal diet (SC)
Kerr et al. (80) (United Kingdom)	Lung cancer	Excluded those with a BMI < 18.5 kg/m^2^	Cancer surgery	I: 33 C: 31	I: 32 C: 29	I: 69.5^§^ C: 71^§^	Hospital- Community	2.4 kcal/ml, 24 En%P cow's milk protein (Fortisip Compact Protein, Nutricia)	600 kcal 36 g P	14 days	3 months	Non-nutritious placebo
Laviano et al. (81) (Italy, Croatia, Slovakia, Sweden)	Lung cancer	Nutritional risk based on weight loss & BMI	Chemotherapy	I: 27 C: 29	I: 25 C: 28	I: 64.4 C: 66.0	Community	1.0 kcal/ml, 20 En%P whey protein 1.0 g omega-3 (0.40 g EPA)/100 ml (ONS name not reported)	400 kcal 20 g P	84 days	12 months	Isocaloric ONS
Lee et al. (82) (Korea)	Colon cancer	Mixed	Cancer surgery	I: 88 C: 88	I: 79 C: 82	I: 65.3 C: 65.3	Community- Hospital	1.0 kcal/ml, 20 En%pt Source of P not available 0.23 g omega-3/100 ml (Newcare Omega, Daesang Life Science)	400 kcal 20 g P	7 days	30 days	Normal diet (SC)
Lee et al. (83) (Korea)	Pancreatobiliary cancer	Mixed	Cancer surgery	I: 30 C: 30	I: 23 C: 18	I: 72.1 C: 73.2	Hospital–Community	51 En%P (ONS name and details not reported)	140 kcal 18 g P	42 days	Not reported	Isocaloric placebo, with no protein
Moya et al. (84) (Spain)	Colorectal cancer	Not malnourished	Cancer surgery	I: 64 C: 64	I: 61 C: 61	I: 69 C: 68	Community–hospital	1.5 kcal/ml 22 En%P source of P not available 0.77 g omega-3/100 ml (IEF-ATEMPERO, Vegenat)	600 kcal 33.2 g P	12 days	30 days	Dietary advice
Pastore et al. (85) (Brazil)	Gastrointestinal and lung cancer	Mixed	Chemotherapy	I: 35 C: 34	I: 28 C: 29	63.5	Community	1.6 kcal/ml, 22 En%P 0.59 g EPA/100 ml (ONS name not reported)	600 kcal 33 g P	28 days	4 weeks	Standard ONS
Ravasco et al. (86) (Portugal)	Head and neck cancer	Mixed	Radiotherapy	I: 25^#^ C: 25	I: 25 C: 25	60	Community	1 kcal/ml, 40 En%P (ONS name not reported)	400 kcal 40 g P	42 days	3 months	Ad lib diet (SC)
			Radiotherapy	I:25^#^ C: 25		60	Community	1 kcal/ml, 40 En%P	400 kcal 40 g P	42 days	3 months	Dietary counselling
Ravasco et al. (87) (Portugal)	Colorectal cancer	Mixed	Radiotherapy	I: 37^##^ C: 37	I: 37 C: 37	58	Community	1 kcal/ml, 40 En%P	400 kcal 40 g P	35 days	3 months	Ad lib diet (SC)
Ravasco et al. (88)^†^ (Portugal)	Colorectal cancer	Mixed	Radiotherapy	I: 37 C: 37	I: 37 C: 37	58	Community	1 kcal/ml, 40 En%P	400 kcal 40 g P	35 days	6.7 years	Ad lib diet (SC)
			Radiotherapy	I: 37 C: 37		60	Community	1 kcal/ml, 40 En%P	400 kcal 40 g P	35 days	6.7 years	Dietary counselling
Sanchez-Lara et al. (89) (Mexico)	Lung cancer	Mixed	Chemotherapy	I: 54 C: 58	I: 46 C: 46	I: 58.8 C: 61	Community	1.2 kcal/ml, 22 En%P cow's milk protein 0.75 g omega-3 (0.45 g EPA)/100 ml (Prosure, Abbott)	590 kcal 32 g P	Not reported	5.8 months	Isocaloric diet (SC)
Sathiaraj et al. (90) (India)	Breast cancer	Not reported	Chemotherapy	I: 52 C: 51	I: 52 C: 51	I: 51 C: 51	Community	3.65 kcal/g 46 En%P whey protein (Kabipro, Fresenius Kabi)	87 kcal 10 g P	84 days	Not reported	Routine care (SC) with dietary advice if malnutrition
Trabal et al. (91) (Spain)	Colorectal cancer	Excluded severe malnutrition (SGA), BMI < 16.5 or >30	Chemotherapy	I: 6 C: 7	I: 5 C: 6	I: 61.5 C: 68.2	Community	1.2 kcal/ml, 22 En%P cow's milk protein 0.75 g omega-3 (0.4 5g EPA)/100 ml (Prosure, Abbott)	590 kcal 32 g P	84 days	12 weeks	Dietary counselling
Ueno et al. (92) (Japan)	Pancreatic cancer	Not reported	Chemotherapy	I: 45 C: 23	I: 43 C: 23	I: 68 C: 69	Community	1.27 kcal/ml, 21 En%P cow's milk protein 0.75 g omega-3 (0.45g EPA)/100 ml (Prosure, Abbott)	560 kcal 29 g P	365 days	12 months	Routine care (SC)
Van der Meij et al. (93) (Netherlands)	Lung cancer	Not reported	Chemoradiotherapy	I: 21 C: 21	I: 20 C: 20	I: 58.4 C: 57.2	Community	1.2 kcal/ml, 22 En%P cow's milk protein 0.75 g omega-3 (0.45 g EPA)/100 ml (Prosure, Abbott)	590 kcal 32 g P	35 days	5 weeks	Standard ONS
Van der Meij et al. (94)^‡^ (Netherlands)	Lung cancer	Not reported	Chemoradiotherapy	I: 21 C: 21	C: 20 C: 20	I: 58.4 C: 57.2	Community	1.2 kcal/ml, 22 En%P cow's milk protein 0.75 g omega-3 (0.45 EPA)/100 ml (Prosure, Abbott)	590 kcal 32 g P	35 days	5 weeks	Standard ONS
Yan et al. (95) (China)	Liver cancer	Not reported	Cancer surgery	I:100 C:100	I: 74 C: 68	I: 56 C: 55	Community-Hospital	1.0 kcal/ml, 20 En%P casein (TP-MCT, Nutricia China)	500-1,000 kcal 25-50 g P	10 days	Not reported To discharge	Routine care (SC)
Zietarska et al. (96) (Poland)	Colorectal cancer	Mixed	Chemotherapy	I: 47 C: 48	I: 47 C: 48	I: 55 C: 63.7	Community	2.4 kcal/ml, 24 En%P (ONS name not reported)	600 kcal 36 g P	84 days	12 weeks	Routine care (SC)

A, analysed; C, comparator/control; E, energy; En%P, % energy from protein; I, intervention; ONS, oral nutritional supplement; P, protein; R, randomised; SC, standard care; Y, years.

^*^Follow-up study of Ida et al. (78).

^†^Follow-up study of Ravasco et al. (87).

^‡^Follow-up study of Van der Meij et al. (93).

^§^Median age.

^¶^Weighted mean age.

^#^Same group of patients.

^##^Same group of patients.

### 3.3 Patients and settings

The total number of patients included in a single study ranged from 13 (91) to 204 (74) (mean 78 patients), with 1,145 patients in total recruited in the intervention group and 1,134 in the control.

#### 3.3.1 Age

The mean or median age of patients ranged from 47 years (70) to 73 years (83). In eleven studies (38%), patients in the intervention group were ≥65 years (*n* = 456) (65, 66, 69, 73, 76, 78, 80, 82–84, 92) and in eighteen studies (62%), patients in the intervention group were ≤ 65 years (*n* = 689) (68, 70–72, 74, 75, 77, 79, 81, 85–87, 89–91, 93, 95, 96).

#### 3.3.2 Setting

In seventeen studies (59%), patients (*n* = 1,100) were in the community only (65, 66, 69–72, 79, 81, 85–87, 89–93, 96) and in eleven studies (38%), patients (*n* = 1,129) were in both community and hospital settings (68, 73–76, 78, 80, 82–84, 95), including community prior to and during hospitalization (68, 73, 74, 82, 84, 95), community prior to, during and following hospitalization (75), and during hospitalisation and post discharge (76, 78, 80, 83). In one study (3%), patients (*n* = 40) were in hospital only (77). Studies were undertaken across many countries and regions of the world, and three studies were multi-country (65, 69, 81) (see Table 2).

#### 3.3.3 Cancers and treatment types

Notably, fourteen studies (48%) (*n* = 1,291) were carried out in patients with GI cancer, specifically upper GI cancer [three studies (71, 76, 78), *n* = 283], lower GI cancer [six studies (68, 82, 84, 87, 91, 96), *n* = 600], pancreatic cancer [four studies (66, 73, 83, 92), *n* = 204] and all GI cancers [one study (74), *n* = 204]. Four studies (*n* = 248) were carried out in patients with lung cancer (80, 81, 89, 93), four studies (*n* = 174) in patients with head and neck cancers (65, 70, 75, 86), two studies (*n* = 182) in patients with liver cancer (77, 95), one study (*n* = 103) in patients with breast cancer (90), and four studies (*n* = 281) in a mix of cancer patients [colorectal and lung cancer (69), colorectal, breast, lung, upper digestive tract, ovarian, and other cancers (72), gastric, colorectal, ovaries, pancreatic, appendix and liver cancer (79) gastrointestinal, and lung cancer (85)]. Sixteen studies (55%) (*n* = 1,008) were conducted in patients undergoing chemotherapy and/or radiotherapy (65, 66, 69–72, 81, 85–87, 89–93, 96) [also including immunotherapy (69)] and thirteen (*n* = 1,271) in patients undergoing cancer surgery (80, 81, 89, 93). Further details of the studies are included in Table 2.

#### 3.3.4 Nutritional status

In twelve studies, both patients with and without malnutrition/nutritional risk were included, so the nutritional status varied (“mixed”) (68, 70, 71, 73, 76, 82, 83, 85–87, 89, 96). In seven studies, nutritional status was not reported (66, 72, 78, 90, 92, 93, 95) and others specifically included [three studies (75, 77, 81)] or excluded [seven studies (65, 69, 74, 79, 80, 84, 91)] patients with malnutrition/nutritional risk (determined by a variety of means, including low body mass index, unplanned weight loss, results of screening tools) (see Table 2 for details).

### 3.4 Intervention and study design

#### 3.4.1 HPONS composition

The HPONS used in the intervention groups had a range of nutritional compositions, with a mean energy density of 1.5 kcal/ml (1.0-3.9 kcal/ml). The ONS energy density was 1 kcal/ml in seven studies (*n* = 861) (74, 76, 81, 82, 86, 87, 95), >1.0 kcal/ml and < 1.5 kcal/ml in ten studies (*n* = 641) (66, 68, 72, 73, 75, 78, 89, 91–93) and ≥1.5 kcal/ml in eleven studies (*n* = 736) (65, 69–71, 77, 79, 80, 84, 85, 90, 96), including seven studies using an ONS >2 kcal/ml (*n* = 408) (65, 69, 70, 77, 80, 90, 96). The ONS energy density was not reported in one study (83). The percentage of energy from protein ranged from 20% (65, 66, 76–78, 81, 82, 95) to 51% (83).

The mean prescribed energy and protein intakes from HPONS were 580 kcal/day (87-1,236 kcal/day) and 35 g/day (10-72 g/day), respectively, based on data from twenty-eight studies [data were not reported in one study (77)]. In eleven of studies (65, 66, 69, 71, 75, 78, 80, 89, 91–93), the HPONS contained cow's milk proteins. Whey protein was the exclusive protein source in six studies (68, 72–74, 81, 90), casein in one study (95) and in one study a mix of cow's milk and plant-based proteins was used (79). Ten studies did not report the protein source (70, 76, 77, 82–87, 96).

The HPONS was enriched with omega-3 fatty acids in sixteen studies (*n* = 1,287), with content ranging from 0.23 g/100 ml to 1 g/100 ml (66, 68, 71–75, 78, 81, 82, 84, 85, 89, 91–93). Eleven out of sixteen studies reported the content of EPA, which ranged from 0.45 mg/100 ml to 0.61 mg/100 ml (66, 71, 72, 75, 78, 81, 85, 89, 91–93).

#### 3.4.2 HPONS format

A ready-to-drink HPONS format was used in twenty-two studies (*n* = 1,669) (65, 66, 69, 71, 75–82, 84–87, 89, 91–93, 95, 96), and a powdered HPONS format was used in six studies (*n* = 569): reconstituted as a liquid in three of these studies (68, 73, 74); as a liquid or mixed with solid food in one study (72) and consumption format was not reported in two of these studies (70, 90). One study (*n* = 41) (83) did not report the format of the ONS used.

#### 3.4.3 Duration of intervention

The duration of intervention with HPONS ranged from 5 days (68, 74) to 365 days (92) with a mean intervention period of 49 days (5-365 days) based on twenty-eight studies [one study (89) did not report the length of intervention]. Most studies (66–82, 84–89, 91–96) included a follow-up period, which ranged from 7 days (77) to 365 days (81, 92). One study (95) did not report the length of follow-up. Two publications reported long-term outcomes of original studies: Aoyama et al. (67) reported long-term survival and surgical morbidity at 3 and 5 years for the cohort in Ida et al. (78) and Ravasco et al. (88) reported survival at 6.5 years for the cohort in Ravasco et al. (87).

#### 3.4.4 Control group

In fifteen out of twenty-nine studies (52%) included in the systematic review, the control group received standard care (SC), which included normal diet, ad lib diet, hospital diet, and isocaloric diet (65, 66, 68, 69, 74–79, 82, 90, 92, 95, 96) of which one study provided dietary advice if malnutrition was detected (90). In five studies (17%), the control group received dietary counselling/advice (DC) (70, 72, 73, 84, 91), of which one used ONS for malnourished patients (73) and one withdrew patients from the DC group if they developed malnutrition (91).

Four studies (14%) used standard ONS (81, 83, 85, 93) as comparator and three studies (11%) reported data for two comparator groups, specifically standard ONS and non-nutritious placebo (stratified according to percentage weight loss: a < 5% weight loss (WL) group supplemented with HPONS vs. non-caloric placebo and a ≥5% WL group supplemented with HPONS vs. standard ONS) (71), and *ad libitum* diet and dietary counselling (86, 87), with both meeting the inclusion criteria for the review and *ad libitum* diet comparator groups selected for inclusion in the meta-analysis. One study (3%) used a non-nutritious placebo (80), and one (3%) isocaloric diet (89) as control.

### 3.5 Quality of studies (*n* = 29)

**Figure 2:**
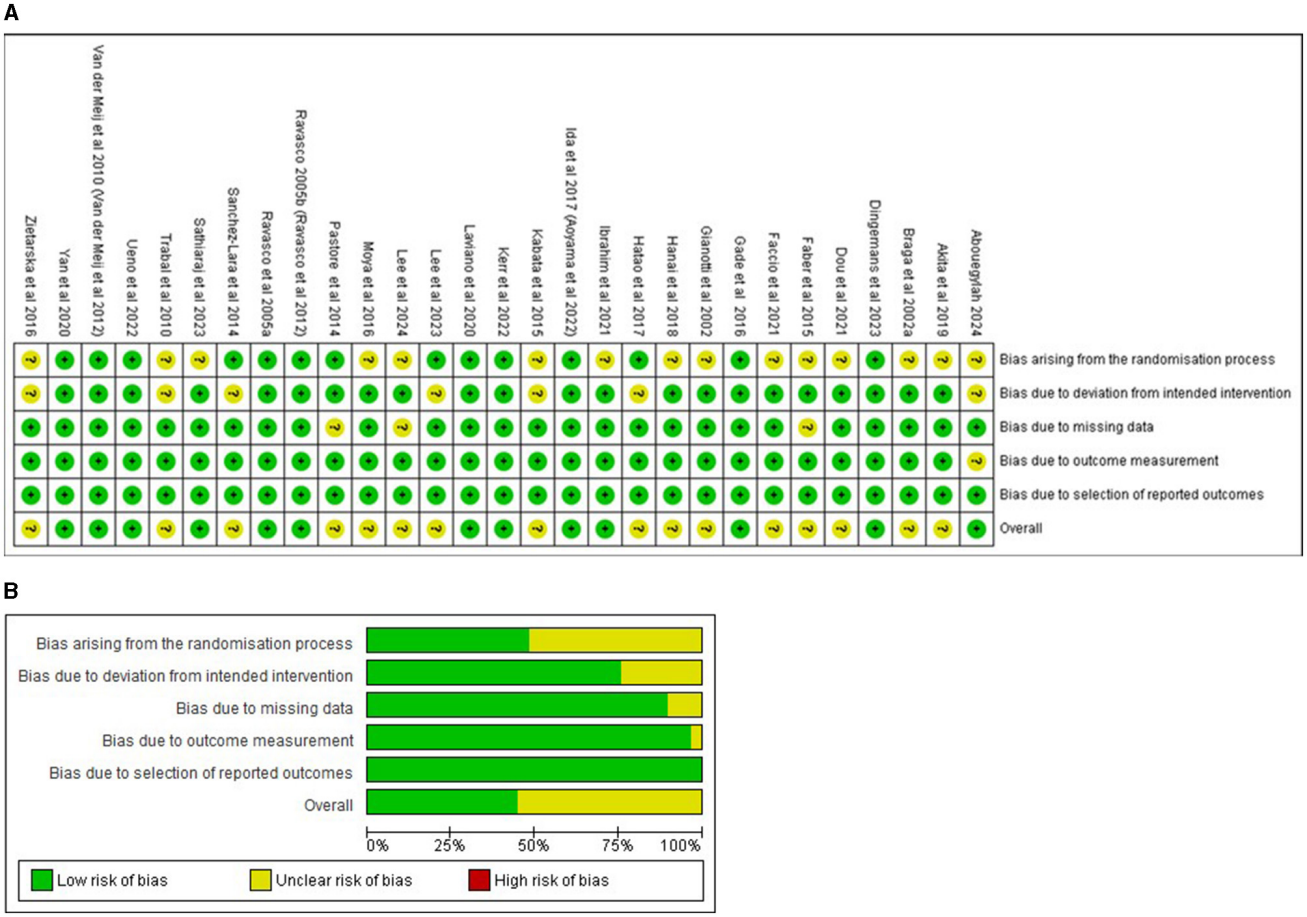
**(a)** Risk of bias summary (RoB2) for included studies (29 RCTs). Assessment of the validity of included studies listed by author and year according to the Cochrane collaborative tool for risk of bias assessment. None of the studies were judged to be at high risk of bias for any of the assessed domains. Colours indicate low + (green), unclear? (yellow) or high – (red) risk of bias. **(b)** Risk of bias graph (RoB2) presented as percentages across all included studies. All included studies are collated for the percentage of risk of bias. Colours indicate low (green), unclear (yellow), or high (red) risk of bias. The overall quality of the included studies was considered adequate.

The risk of bias (RoB2) assessment across the five domains is summarised below and presented in Figures 2a, b.

All twenty-nine studies were at low risk of bias due to the selection of reported outcomes. None of the studies were judged to be at high risk of bias for any of the assessed domains.

Deviations from the PICO criteria were not identified; thus, all studies were judged to be directly applicable.

Of the twenty-nine unique RCTs, 10 were judged to be of a low risk of overall bias (69, 73, 78, 80, 81, 86, 87, 92, 93, 95).

The remaining nineteen studies raised some concerns of bias in one or more domains. Fifteen studies did not adequately report on the randomisation process (65, 66, 68, 70–72, 74, 75, 77, 79, 83, 84, 90, 91, 96).

Seven studies were judged to have some concern of bias due to deviations from intended interventions (65, 76, 79, 83, 89, 91, 96), three studies raised some concerns of bias due to missing outcome data (71, 83, 85) and one study raised some concerns of bias in outcome measurement (65).

Publication bias was assessed in the meta-analysis with ≥10 pooled RCTs pertaining to complications. The visual inspection of the funnel plot (not shown) did not suggest the presence of substantial asymmetry, indicating an absence of publication bias; considering the low between-study heterogeneity, the scatter observed could be attributed to sampling variations.

The certainty of evidence by GRADE assessment across pooled outcomes ranged from very low to moderate, and the downgrading was mainly due to the presence of serious risk of bias and/or serious imprecision across the pooled studies.

### 3.6 Outcomes

#### 3.6.1 Complications

Complications were reported in seventeen studies (*n* = 1,383) (65, 68, 73–75, 77–82, 84, 88, 91, 92, 94, 96). Fifteen studies were pooled for meta-analysis (65, 68, 73–75, 77–80, 82, 84, 88, 91, 92, 96), with two studies analysed separately as they used standard ONS as control (81, 94). Complications reported in each study are summarised in Table 3. Five studies reported infectious complications (68, 73, 74, 78, 79), four non-infectious complications (68, 73, 74, 82), three surgical complications (79, 80, 84), four chemo/radiotherapy-related toxicities (88, 91, 92, 96), and two post-operative wound complications (75, 80). Ten studies were conducted in patients with GI cancer (68, 73, 74, 78, 82, 84, 88, 91, 92, 96), two studies in patients with head and neck cancer (65, 75), and one study in patients with each of the following cancers: liver (77), lung (80), and mixed (79).

**Table 3:** Summary of complications reported in each study (*n* = 17) arranged according to first author's surname.

**Study**	**Complications**
Abouegylah et al. (65)	- Chemo/radiotherapy related toxicity (oral mucositis^*^, xerostomia^*^, nausea, dysphagia, dermatitis)
	^*^ *n of events used in meta-analysis as n of total complications not being reported*
Braga et al. (68)	-Post-operative infections (respiratory tract, wound, urinary tract, bacteraemia, abdominal abscess
	-Non-infectious complications (pleural effusion/atelectasia, respiratory insufficiency, cardiac failure, bleeding, deep vein thrombosis, renal dysfunction, intestinal obstruction, wound dehiscence, anastomotic leak)
Gade et al. (73)	-Post-operative infections (septic shock, sepsis, systemic inflammatory response syndrome, anastomotic leak, intra-abdominal abscess, cholangitis, pneumonia, local wound infection, fungal infection, infectious diarrhoea)
	-Non-infectious complications (vascular insufficiency, cardiac insufficiency, cardiac arrhythmia, hypovolemia, multiorgan dysfunction syndrome, renal insufficiency, ileus, chylous, fistula, abdominal bleeding, bleeding from cicatrice, respiratory insufficiency, atelectasis, transient ischemic attack, venous thrombosis, non-infectious wound complication, non-infectious diarrhoea, anaemia)
Gianotti et al. (74)	-Post-operative infections (wound, abdominal abscess, respiratory tract, urinary tract, bacteraemia, sepsis)
	-Non-infectious complications (respiratory failure, delayed gastric emptying, pancreatic fistula, circulatory insufficiency, bleeding, wound dehiscence, pleural effusion, renal dysfunction, intestinal obstruction, pulmonary embolism, anastomotic leak)
Hanai et al. (75)	-Post-operative complications (wound complication)
Ibrahim et al. (77)	-Infectious complications (*no additional details reported*)
Ida et al. (78)	-Post-operative infections (pancreatic fistula, abdominal abscess, leakage, bleeding, other unspecified complications)
Kabata et al. (79)	-Infectious complications (wound infection, pneumonia, sepsis)
	-Surgical complications (subileus, mechanical ileus, gastric bleeding, oesophageal graft perforation, anastomotic leakage, evisceration)
	-General complications (fluid-electrolyte disturbances, cardiac-neurologic)
Kerr et al. (80)	-Post-operative complications (pulmonary complications, wound complications)
Laviano et al. (81)	-Infectious complications (Pneumonia)
	-Non-infectious complications (disease progression, neoplasms benign, malignant, and unspecified, vascular, blood and lymphatic system disorders, pulmonary embolism, toxicity to various agents, GI disorders)
	-Chemotherapy-dose limiting toxicities^*^
	^*^ *n of events used in meta-analysis*
Lee et al. (82)	-Infectious complications (wound infection, organ-space surgical site infection, urinary tract infection, Clostridium difficile infection, pneumonia),
	-Non-infectious complications (prolonged post-operative ileus, post-operative urinary retention, cardiovascular complications, delirium)
Moya et al. (84)	-Surgical complications (anastomotic leak, paralytic ileus, other unspecified surgical complications)
	Infectious complications (wound infections, respiratory infections, venous catheter infections)
Ravasco et al. (88)	-Radiotherapy toxicity (acute radiotherapy toxicity, late radiotherapy toxicity including flatulence, abdominal distension, diarrhoea)
Trabal et al. (91)	-Chemotherapy-related toxicities (*no additional details reported*)
Ueno et al. (92)	-Infectious complications (mainly cholangitis)
Van der Meij et al. (94)	-Chemotherapy-related toxicities (chemotherapy delays, chemotherapy dose reduction)
Zietarska et al. (96)	-Infectious complications (sepsis)
	-Chemotherapy-related toxicities (*no additional details reported)*

Ten studies involved patients undergoing surgery for cancer (68, 73–75, 77–80, 82, 84) and five studies involved patients undergoing chemotherapy (91, 92, 96), radiotherapy (88), or chemo/radiotherapy (65). In six studies (65, 79, 88, 91, 92, 96) supplementation was carried out entirely in the community (65, 79, 88, 91, 92, 96), in five studies HPONS were initiated in the community prior to surgery and continued in hospital (68, 73, 74, 82, 84), in two studies (77, 78) supplementation was carried out entirely in hospital (77, 78), in one study supplementation was initiated in hospital post-surgery and continued post-discharge in the community (80) and in one study supplementation was initiated prior to surgery in the community and continued in hospital and post-discharge (75).

The two studies that compared HPONS versus standard ONS were conducted in patients with lung cancer undergoing chemotherapy in the community setting (81, 94). These studies assessed chemotherapy-related toxicity (81, 94) and infectious & non-infectious complications (81).

**Figure 3:**
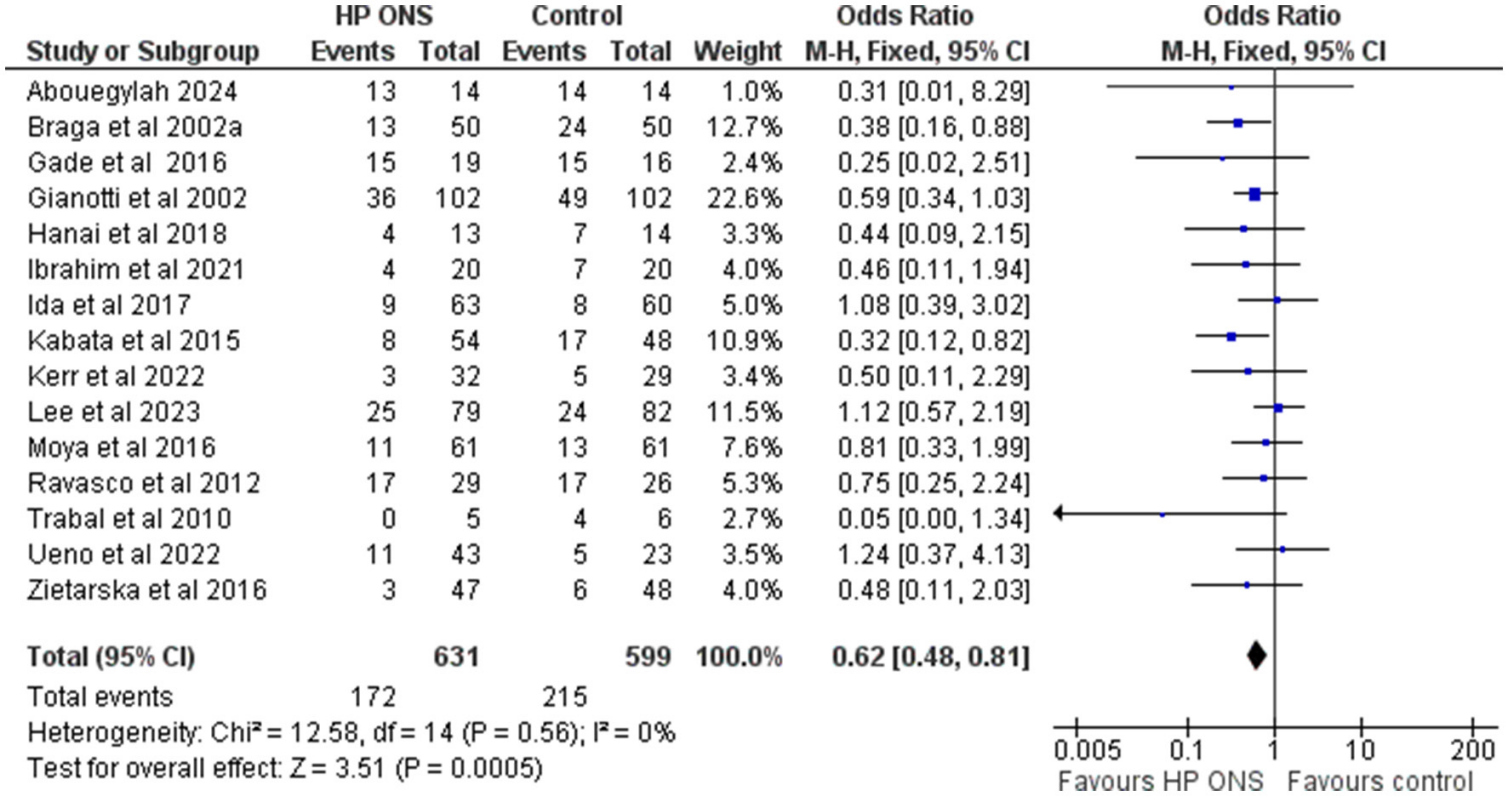
Fixed-effects meta-analysis of RCT using HPONS in the intervention group (*n* = 15) reporting complications.

A meta-analysis of fifteen studies [*n* = 1,230; GI cancers ten studies (68, 73, 74, 78, 82, 84, 88, 91, 92, 96); head and neck cancers two studies (65, 75); other three studies (77, 79, 80)], undergoing surgery [ten studies (68, 73–75, 77–80, 82, 84)] or chemotherapy and/or radiotherapy [five studies (65, 88, 91, 92, 96)] showed that patients receiving HPONS had a significantly reduced incidence of complications compared to the control group (OR: 0.62, 95% CI: 0.48-0.81; *p* = 0.0005; *I*^2^ = 0%), equivalent to 101 (from 147 to 47) fewer events per 1,000 patients (see Figure 3). The NNT for preventing one additional complication with HPONS compared to control was 12 (95% CI: 9-29) (see Table 4).

**Table 4:** Summary of findings table for complications comparing HPONS vs. control.

**Participants (studies)**	**Certainty of the evidence (GRADE)**	**Study event rates (%)**		**Anticipated absolute effects** ^*^		
		**With control or standard diet**	**With HPONS**	**Relative effect (95% CI)**	**Risk difference with HPONS**	**NNT (95% CI)**
**Complications: HPONS vs. control**						
1,230 (15 RCTs)	⊕⊕○○ Low^a, b^	215/599 (35.9%)	172/631 (27.3%)	OR: 0.62 (0.48–0.81)	101 fewer per 1,000 (from 147 to 47 fewer)	12 (8–29)
**Complications: HPONS containing omega-3 vs. control**						
849 (9 RCTs)	⊕⊕○○ Low^a, b^	149/414 (36.0%)	124/435 (28.5%)	OR: 0.69 (0.51–0.93)	80 fewer per 1,000 (from 137 to 17 fewer)	14 (8–83)
**Complications: HPONS without omega-3 vs. control**						
381 (6 RCTs)	⊕⊕⊕○ Moderate^a^	66/185 (35.7%)	44/198 (24.2%)	OR: 0.46 (0.27–0.79)	153 fewer per 1,000 (from 227 to 52 fewer)	9 (5–49)

CI, confidence interval; HPONS, high-protein oral nutritional supplement; NNT, number needed to treat; OR, odds ratio; RCT, randomised controlled trial.

^*^The risk in the intervention group (and its 95% confidence interval) is based on the assumed risk in the comparison group and the relative effect of the intervention (and its 95% CI).

GRADE Working Group grades of evidence. High: we are very confident that the true effect lies close to that of the estimated effect. Moderate: we are moderately confident in the effect estimate: the true effect is likely to be close to the estimate of the effect, but there is a possibility that it is substantially different. Low: our confidence in the effect estimate is limited; the true effect may be substantially different from the estimate of the effect. Very low: we have very little confidence in the effect estimate; the true effect is likely to be substantially different from the estimated effect.

^a^>33.3% of the weight in a meta-analysis came from studies at moderate or high risk of bias.

^b^Downgraded one level for imprecision, as the 95% confidence interval for the effect size crosses one line of the clinical decision threshold.

**Figure 4:**
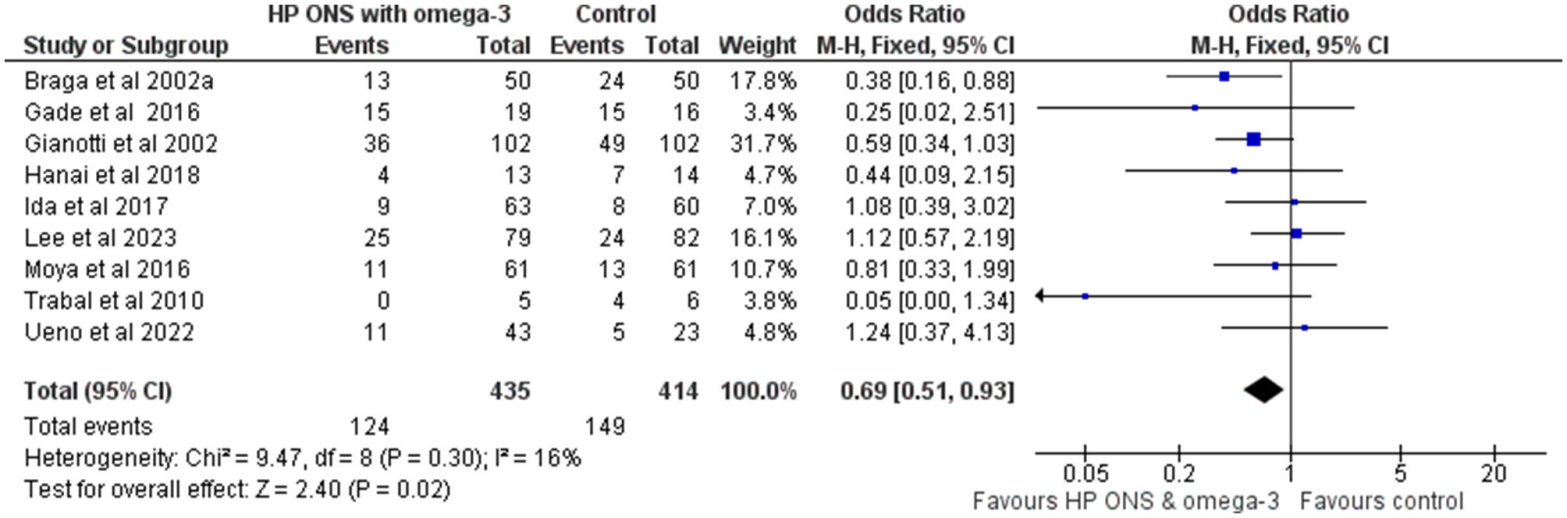
Fixed-effects meta-analysis of RCT using a high-protein ONS containing omega-3 in the intervention group (*n* = 9) reporting complications.

Nine of the fifteen studies (60%) (*n* = 849) used an HPONS containing omega-3 in patients with GI cancers (68, 73, 74, 78, 82, 84, 91, 92) and head and neck cancers (75) undergoing surgery [seven studies (68, 73, 74, 78, 82, 84)] or chemotherapy [two studies (91, 92)]. An exploratory meta-analysis was undertaken and showed a significantly reduced incidence of complications in this sub-group compared to control (OR: 0.69, 95% CI: 0.51-0.93; *p* = 0.02; *I*^2^ = 16%) equivalent to 80 (from 137 to 17) fewer events per 1,000 patients (see Figure 4). The NNT for preventing one additional complication with HPONS containing omega-3 compared to control was 14 (95% CI: 8-83) (see Table 4).

**Figure 5:**
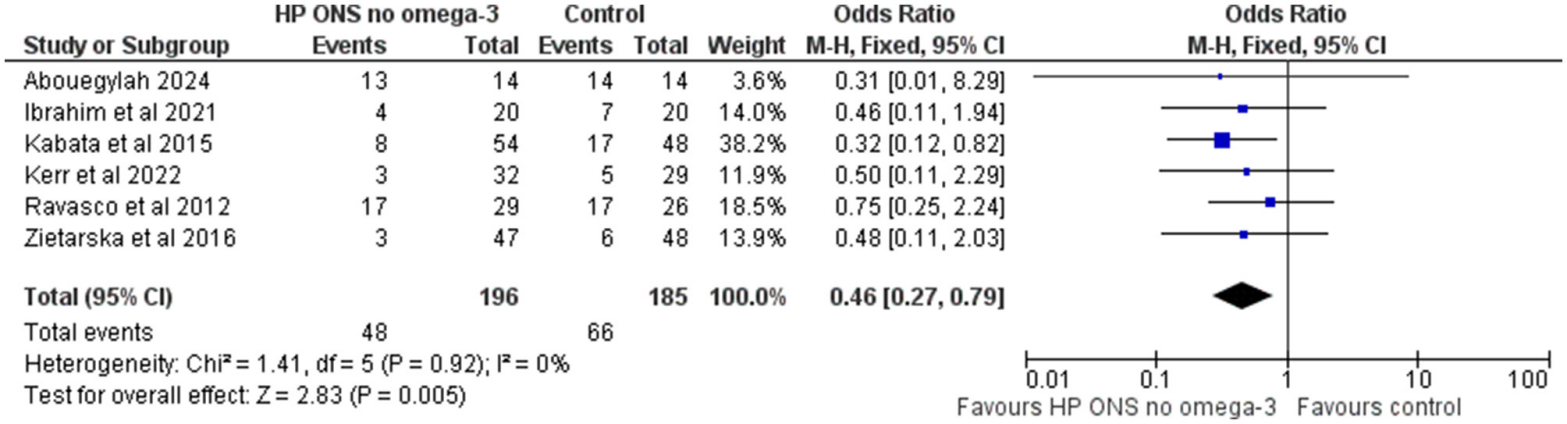
Fixed-effects meta-analysis of RCT using a high-protein ONS without omega-3 in the intervention group (*n* = 6) reporting complications.

Similarly, in the sub-group of six studies (40%) (*n* = 381) using an HPONS without omega-3, in patients with GI cancers (79, 88, 96), liver cancer (77), head and neck cancer (65), and lung cancer (80), undergoing surgery [three studies (77, 79, 80)] or chemotherapy and/or radiotherapy [three studies (77, 79, 80)], the exploratory meta-analysis showed a significant reduction in the incidence of complications compared to the control (OR: 0.46, 95% CI: 0.27-0.79; *p* = 0.005; *I*^2^ = 0%) equivalent to 153 (from 227 to 52) fewer events per 1,000 patients (see Figure 5). The NNT for preventing one additional complication with HPONS without omega-3 was 9 (95% CI: 5-49) (see Table 4).

Summary of meta-analysis outcomes, risk difference and NNT for complications are presented in Table 4 and Supplementary material.

A separate meta-analysis was conducted by pooling the two studies (81, 94) (*n* = 95) that compared the incidence of complications in patients using HPONS vs. standard ONS. The pooled effect estimate showed no significant difference in the occurrence of complications between the two groups (OR: 1.20, 95% CI: 0.43 to 3.37; *p* = 0.72; I^2^ = 0%).

#### 3.6.2 Length of hospital stay

Length of hospital stay was reported in eight studies (*n* = 864) (68, 73, 74, 77, 80, 82, 84, 95) and were pooled into meta-analysis. The studies involved patients undergoing surgery for GI (68, 73, 74, 82, 84), liver (77, 95), and lung (80) cancers. In six of these studies, supplementation with HPONS was initiated in the community prior to surgery and continued in hospital (68, 73, 74, 82, 84, 95), in one study it was initiated in hospital post-surgery and continued post-discharge in the community (80), and in one study supplementation was initiated in the community pre-surgery and continued during hospitalization (82). Five of these studies (63%) (*n* = 621) used an HPONS containing omega-3 (68, 73, 74, 82, 84), and three studies (*n* = 243) used an HPONS without omega-3 (68, 73, 74, 82, 84).

**Figure 6:**
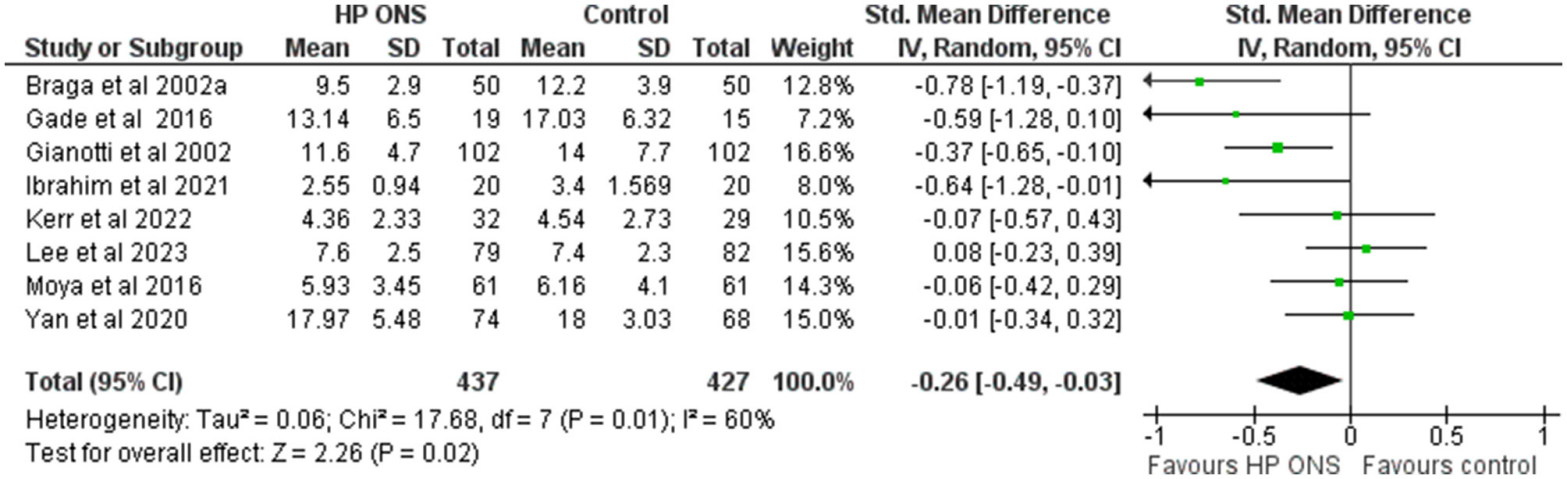
Random-effects meta-analysis of RCT (*n* = 8) reporting length of hospital stay.

A random-effects meta-analysis of the eight studies (*n* = 865) was undertaken and showed patients receiving an HPONS had a significantly lower LOS vs. control (SMD: −0.26, 95% CI: −0.49 to −0.03; *p* = 0.02, *I*^2^ = 60%) (Figure 6). Sensitivity analysis was carried out (*n* = 505) by removing the four studies (73, 80, 84, 95) that reported median [interquartile range (IQR)] length of hospitalisation, which were subsequently converted to mean (SD). The effect estimate favoured HPONS relative to control [−0.39, 95% CI: −0.78 to −0.01; *p* = 0.0]. Sub-group meta-analysis of HPONS trials with and without omega-3 fatty acids showed no significant effects. Summary of meta-analysis findings is presented in Table 5 and in Supplementary material.

**Table 5:** Summary of findings table for length of hospital stay, hospital readmissions and mortality comparing HPONS vs. control.

**Participants (studies)**	**Certainty of the evidence (GRADE)**	**Study event rates (%)**		**Anticipated absolute effects**	
		**With control or standard diet**	**With HPONS**	**Relative effect (95% CI)**	**Risk difference with HPONS**
**Length of hospital stay**					
864 (8 RCTs)	⊕○○○ Very low^a, b, c^	–	–	–	0.26 days lower^*^ (0.49–0.03)
**Hospital readmissions**					
479 (5 RCTs)	⊕○○○ Very low^a, d^	24/238	25/241	OR: 1.02 (0.56–1.87)	2 more per 1,000 (from 42 fewer to 73 more)
**Mortality**					
694 (7 RCTs)	⊕⊕○○ Low^a, b^	59/334	62/360	OR: 0.68 (0.42–1.11)	49 fewer per 1,000 (from 94 fewer to 16 more)

^*^Standardised mean difference.

GRADE Working Group grades of evidence.

High: we are very confident that the true effect lies close to that of the estimated effect. Moderate: we are moderately confident in the effect estimate: the true effect is likely to be close to the estimate of the effect, but there is a possibility that it is substantially different. Low: our confidence in the effect estimate is limited; the true effect may be substantially different from the estimate of the effect. Very low: we have very little confidence in the effect estimate; the true effect is likely to be substantially different from the estimated effect.

^a^>33.3% of the weight in a meta-analysis came from studies at moderate risk of bias.

^b^Downgraded one level for imprecision, as the 95% confidence interval for the effect size crosses one line of the clinical decision threshold.

^c^Downgraded one level for inconsistency >33.3%.

^d^Downgraded two levels as 95% confidence intervals for the effect size cross both lines of the clinical decision threshold.

#### 3.6.3 Readmissions to hospital

Readmissions to hospital were reported in six studies (*n* = 519) (68, 73, 80, 82, 84, 94), within 30 days post-discharge (68, 73, 82, 84), 5 weeks (94) and 3 months follow-up (80). Five studies (68, 73, 80, 82, 84) were pooled into meta-analysis, with one (94) excluded as it used standard ONS as control.

The studies involved patients undergoing surgery for GI (68, 73, 82, 84) and lung (80) cancers. Supplementation with an HPONS containing omega-3 was initiated in the community prior to surgery and continued during hospital stay in four studies (68, 73, 82, 84), and supplementation with an HPONS without omega-3 was initiated in hospital post-surgery and continued post-discharge in the community in one study (80).

A meta-analysis of the five RCTs (*n* = 479) revealed no significant difference in the proportion of patients receiving HPONS readmitted to hospital compared to the control group (OR: 1.02, 95% CI: 0.56-1.87; *p* = 0.94; *I*^2^ = 0%). Van der Meij et al. (94), that was excluded from meta-analysis, showed no difference in non-scheduled hospital admissions between groups (p=0.87). A summary of the meta-analysis findings is presented in Table 5 and in Supplementary material.

#### 3.6.4 Mortality

Measures of mortality were reported in ten studies (*n* = 969), with numbers of deaths within the intervention and follow-up period (30 days to 12 months) reported in seven studies (68, 73, 74, 80, 81, 84, 89) and additional measures of survival during follow-up (1 month to 6.7 years) were used to extrapolate mortality data in three studies (67, 88, 92). No events were observed in both intervention arms of Kerr et al. (80) and Moya et al. (84), hence these studies were excluded from this meta-analysis. One study (81) compared HPONS vs. standard ONS and was excluded from meta-analysis.

Six studies were conducted in patients with GI cancer (67, 68, 73, 74, 88, 92), and one study in lung cancer patients (89).

Four studies involved patients undergoing cancer surgery (67, 68, 73, 74), and three studies involved patients undergoing chemo/radiotherapy (88, 89, 92). The HPONS supplementation was initiated in the community prior to surgery and continued during hospital stay in three studies (68, 73, 74), and was initiated in hospital post-surgery and continued post-discharge in one study (80). Supplementation was carried out entirely in the community in three studies (88, 89, 92) and entirely in a hospital in one study (67).

Six studies used an HPONS containing omega-3 (67, 68, 73, 74, 89, 92), and one study used an HPONS without omega-3 (88).

A meta-analysis of the seven studies (*n* = 694) (67, 68, 73, 74, 88, 89, 92) was undertaken using a fixed-effect model and showed no significant difference in mortality in patients receiving an HPONS compared to the control group (OR: 0.68, 95% CI: 0.42-1.11; *I*^2^ = 0; *p* = 0.12). A summary of meta-analysis findings is presented in Table 5 and Supplementary material.

#### 3.6.5 Compliance

Relevant measures of compliance to the HPONS (% of HPONS consumed vs. prescribed) were reported in nineteen studies (*n* = 1,434) (68–78, 80–84, 89, 91, 93). One study, which aimed to evaluate compliance as its primary outcome, (85) was excluded from the descriptive summary of compliance data as it reported HPONS discontinuation rates rather than actual intake relative to the prescribed dose.

Eight studies (*n* = 659) (ONS energy density from 1.0 to 2.4 kcal/ml) reported ≥80% compliance during the intervention period (68, 71, 73, 74, 77, 80, 82, 91). Six studies were conducted in patients with GI cancers (68, 71, 73, 74, 82, 91), one in liver cancer (77), and one in lung cancer (80). Most studies (six out of eight) involved patients undergoing surgery (68, 73, 74, 77, 80, 82) and two studies were conducted in patients undergoing chemo and chemoradiotherapy (71, 91). In four studies, supplementation with HPONS was initiated in the community prior to surgery and continued in hospital (68, 73, 74, 82), was initiated in hospital post-surgery and continued post-discharge in the community in one study (65, 87, 88, 91, 92, 96), supplementation was carried out entirely in hospital in one study (77), and entirely in the community in two studies (91). Six of these studies (71, 73, 74, 82, 91) used an HPONS (1.0–1.6 kcal/ml) containing omega-3 (68, 71, 73), (74, 82, 91).

Eight studies (*n* = 585) (ONS energy density from 1.0 to 2.4 kcal/ml) reported compliance < 80% during the intervention period (69, 72, 76, 81, 83, 84, 89, 93). Three studies were conducted in GI cancer (76, 83, 84), three in lung cancer (81, 89, 93), and two in mixed cancers (69, 72) patients. Most studies (five out of eight) involved community patients undergoing chemo and chemoradiotherapy (69, 72, 81, 89, 93), and three studies involved patients undergoing surgery (76, 83, 84). Five out of eight studies used an HPONS containing omega-3 (72, 81, 84, 89, 93).

Three studies reported changes in compliance over time, specifically two studies reported compliance ≥80% pre-surgery, then decreasing to < 80% in post-surgery (75, 78) cancer patients, and one study reported ≥80% (87.2%−89.5%) compliance in the first 2 weeks of intervention in community patients undergoing chemotherapy, with a decrease (40.5%−63.6%) in the following 4 weeks (70).

#### 3.6.6 Energy and protein intake

Eleven studies (*n* = 684) reported mean total energy intake data, with substantial heterogeneity in how this was presented (66, 69, 72, 75, 83, 86, 87, 89–91, 93), and five of these studies reported an increase in energy intake in the HPONS group (72, 86, 87, 89, 93) compared to baseline, of which three reported a statistically significant increase (72, 86, 87, 89, 93). In contrast, energy intake declined in the control groups in six of these studies (66, 72, 86, 87, 89, 93), with two reporting statistically significant reductions (86, 87). One of these studies in patients with breast cancer reported a statistically significant decrease in energy intake over time in the HPONS group (using a powder ONS) compared to baseline (90).

Between-group comparisons showed higher total energy intake in the HPONS group than the control group across nine studies (69, 72, 75, 83, 86, 87, 89, 91, 93), with six of these studies using ready-made ONS (all in patients undergoing chemotherapy and/or radiotherapy) reporting statistically significant differences (69, 72, 86, 87, 89, 93), three of which were with HPONS [omega-3 (72, 89, 93)]. One study (66) reported no difference in energy intake between the HPONS and control group at the end of the intervention period, and one study using a powder ONS in patients with breast cancer reported lower energy intake in the HPONS group vs. control (90).

Eight studies (*n* = 595) reported total protein intake data, with substantial heterogeneity in how this was presented (69, 72, 83, 86, 87, 89, 90, 93), with seven of these studies (69, 72, 86, 87, 89, 90, 93) reporting increased protein intake in the HPONS group at the end of intervention compared to baseline, five of which (72, 86, 87, 89, 90) were statistically significant. In contrast, control groups showed mixed results, with three studies reporting reduced protein intake (86, 87, 89) and other studies reporting increases vs. baseline (69, 72), including one in which the control group received standard ONS (93).

Between-group comparisons showed significantly higher total protein intake in the HPONS group (using ready-made formats) than the control group in six studies (69, 72, 83, 86, 87, 89), mostly (five out of six) in patients undergoing chemotherapy and/or radiotherapy, two of which were with an HPONS with omega-3 (72, 89). One study reported that only the HPONS group met nutritional requirements, though without statistically significant between-group differences (91). One study comparing HPONS with a standard ONS in lung cancer patients undergoing chemoradiotherapy found no difference in total protein intakes between groups (93).

## 4 Discussion

This systematic review of twenty-nine studies (in 2,279 patients, 1,145 receiving HPONS) comprehensively explored the effects of using HPONS (both short and long term) on clinical outcomes in patients with a range of cancers undergoing a variety of treatment modalities across hospital and community settings.

A major finding of this systematic review and meta-analysis was a significant overall reduction (101 fewer per 1,000 patients) in complications, including infectious and non-infectious complications, post-operative and chemo/radiotherapy-related complications. The NNT indicated that, on average, treating 12 patients with HPONS would prevent one additional complication compared to control. In our meta-analyses, significant reductions in complications were observed with the use of HPONS (including those with and without omega-3) in addition to the diet, which supports the recommendations made by international societies, such as ESMO and ESPEN (18, 19, 21). Overall, most studies in the meta-analyses of HPONS were in patients with GI cancers (colon/rectal, pancreas, and other GI) undergoing surgery, mostly patients were not specifically identified as malnourished/at nutritional risk (typically patients included had a range of nutritional status), and the control group typically received routine care. Interventions ranged from very short periods of time pre-operatively in those undergoing surgery (5 days) to long community-based nutritional support during chemo/radiotherapy (up to 1 year) as clinically indicated. Specifically, in the studies of HPONS containing omega-3, the majority (eight out of nine studies) were conducted in patients with gastrointestinal cancers (colorectal, pancreatic, or mixed types). Most of these studies focused on surgical or post-operative complications, both infectious and non-infectious, while one study (91) reported chemotherapy-related toxicity in colorectal cancer patients. The meta-analysis of studies using HPONS without omega-3 included a broader range of cancer types. These studies reported a variety of complications, including chemotherapy- and radiotherapy-related toxicities, as well as post-surgical complications, both infectious and non-infectious, among patients with different nutritional statuses across hospital and community settings. The heterogeneity in cancer types, treatments, settings, and nutritional status of included populations is a critical factor influencing the interpretation and generalisability of findings. While the strongest evidence for benefit was found in GI cancers, particularly those undergoing surgery, the significant effects observed in studies involving other cancer types suggest that HPONS may offer broader utility. In our sub-group analysis, both HPONS with and without omega-3 were associated with reduced complications, though the composition and clinical context varied, with HPONS without omega-3 tested in more heterogeneous cancer types. These findings imply that HPONS may have efficacy across multiple scenarios, suggesting that further stratified research is necessary.

The reduction in complications associated with HPONS observed in this review aligns with findings from an earlier systematic review and meta-analysis, which evaluated the effects of ONS on complications across diverse patient populations in community settings (97). That earlier review indicated an NNT of 14 patients overall and highlighted a significant reduction in both infectious and non-infectious complications in surgical and medical patients receiving HPONS (OR: 0.66, 95% CI: 0.54-0.80, *p* < 0.01, *n* = 2,826) across many patient groups, including those with cancer. Another recent systematic review in patients undergoing gastrointestinal surgery for cancer indicated a NNT of seven and NNT of eight patients, highlighting pre-operative ONS reduced all cause post-operative surgical complications (RR: 0.53, 95% CI: 0.46–0.60, *p* < 0.001, *n* = 891), and infection (RR: 0.52, 0.40–0.67, *p* = 0.008, *n* = 570), respectively (30).

Other reviews of studies in patients with cancer that assessed the impact of HPONS or other nutritional interventions on complications have shown mixed results, due to differences in included patient populations, the composition, and/or timing of the interventions used and the presence of concomitant treatments/surgery.

Orsso et al. (49) reported that high-protein supplementation (using a different definition of >10 g protein per serving) was associated with reduced cancer therapy-induced toxicity in 57% of studies, though results varied by supplement type and treatment context. While evidence was mixed, high-protein intervention showed potential to improve chemotherapy tolerance and response, with some studies reporting benefits on treatment modifications and clinical outcomes. Another recent meta-analysis suggested that usage of whey protein exclusively or in conjunction with other interventions in the perioperative period significantly decreased post-operative complications in patients with gynaecological cancer (42). On the contrary, another systematic review of pre-operative oral nutritional support in patients undergoing colorectal surgery reported no significant reduction in overall complication rate in patients taking ONS. However, the studies were heterogeneous, and in one of the five studies analyzed, patients did not use an HPONS (98).

In terms of healthcare use, this systematic review and meta-analysis found that the use of HPONS significantly reduced length of stay in patients with cancer who underwent surgery (mostly patients undergoing surgery for cancers of the GI tract), although no significant effect on hospital readmissions was found. The reduction in length of stay could be attributed to the reduction in complications found in the peri-operative period, leading to patients recovering more quickly (and with no significant differences in mortality rates found between groups). Changes in other patient outcomes that may have contributed to a quicker recovery (muscle strength, mobility, quality of life, etc.), observed in other reviews of nutritional interventions, including HPONS (41) could have led to shorter stays, but are beyond the scope of this current review. Reductions in hospital length of stay with ONS have been previously reported in systematic reviews of trials in a range of patient populations, including those with cancer (41, 99, 100). Specifically, pre- and post-operative HPONS use was shown to reduce length of stay in patients with colorectal cancer regardless of initial nutritional status, which resulted in significantly lower treatment costs during hospitalisation and at 6 months after surgery (101).

Although other systematic reviews have shown reductions in hospital readmissions with the use of HPONS in a range of patient groups (not limited to cancer) (97, 102), in this review, the short intervention period (5-14 days) with HPONS in patients undergoing surgery may explain why no significant differences were observed. Indeed, numerous factors in cancer patients undergoing surgery, such as underlying health conditions, LOS, post-discharge care (and availability) and adherence to nutritional recommendations, could also influence hospital readmissions (103). A longer intervention period might be necessary to fully capture the potential benefits of HPONS on hospital readmission rates, but further research is needed to help clarify these relationships and optimise nutritional support strategies.

Similarly, no significant differences in mortality were observed with the use of HPONS compared to controls, based primarily on studies conducted in community settings, although Ueno et al. reported higher 1-year survival in patients undergoing chemotherapy for pancreatic cancer receiving omega-3-enriched HPONS (92). This contrasts with one recent randomised trial in hospital patients with various cancer types that found diverse forms of nutritional support significantly reduced mortality (43).

Interventions such as HPONS, that contribute to improved outcomes, as the reduction of complications and reduced healthcare use, may also be beneficial for reducing healthcare costs. By preventing complications, HPONS can potentially decrease the need for additional treatments, prolonged hospital stays, and other associated expenses, leading to significant cost savings for healthcare systems. A reduction in the length of hospital stay has important economic implications; the average cost of a hospital stay per day in the UK is approximately GBP £345 for a standard bed and as high as GBP £2,349 for elective care (104). Reducing the length of stay by even a few days can result in substantial savings, highlighting the economic value of effective nutritional interventions. The financial burden associated with complications in cancer care is also substantial and consistently demonstrated across diverse healthcare settings and cancer types. Evidence indicates that complications significantly escalate direct medical costs, largely due to increased hospitalisations, intensive interventions, and prolonged recovery times (105–110). A formal economic evaluation using the clinical outcomes in this review will subsequently be undertaken to elucidate the cost-effectiveness of HPONS in this patient group.

The mechanism by which the use of a multi-nutrient, HP liquid supplement may reduce complications and LOS in patients with cancer undergoing treatment or surgery was not investigated in the studies included in this systematic review. It is also unlikely that a single mechanism accounts for the observed effects of HPONS, as outcomes are likely influenced by multiple factors. These include the specific characteristics of the supplement (e.g., energy density, protein type and quantity, micronutrient composition, timing, duration, and patient compliance), the nature of the therapeutic interventions (e.g., type and intensity of chemotherapy, radiotherapy, or surgical procedures), and the cancer's location, stage, and type.

However, what is common to all the included studies is the provision of a range of additional nutrients into the body from the intake of HPONS, which is likely to play a fundamental role in improving outcomes. These benefits may stem from the intake of energy, protein, and other key nutrients that help preserve or restore body reserves, such as body weight and fat-free mass (primarily muscle), which are often compromised due to cancer-related symptoms and the side effects of treatment. Maintaining these reserves may enhance patients' ability to tolerate and recover from chemotherapy, radiotherapy, and surgery. Additionally (either independently or interrelated), it may be the impact of one or more macronutrients (including individual amino acids, or specific fatty acids such as EPA or DHA) or micronutrients on the function of tissues or cells that impact the inflammatory and immune response and response to healing. Specifically, evidence shows that adequate protein intake is essential for the synthesis of immune cells and antibodies, which can help in preventing infections and favouring the healing process by promoting collagen synthesis and tissue regeneration. The reduction in complications may be attributed to the improved nutritional status provided by HPONS, which supports recovery and reduces the risk of other complications such as infections and delayed wound healing. Similarly, in patients undergoing radiotherapy, HPONS may mitigate the adverse effects of treatment by maintaining muscle mass and overall nutritional status, thereby reducing treatment-related complications (111, 112).

To explore this further, this systematic review also investigated the impact of HPONS on nutritional intakes, looking at any data on compliance with ONS and total nutritional intakes (from the diet plus the intervention). Compliance (where recorded) ranged from 40.5% to 100%, a broad range similar to what was reported in previous reviews of trials of compliance across different patient groups and settings and specifically among patients with cancer (ranged from 6.0% to 96.9%), with patients taking HPONS showing higher adherence (113).

In this systematic review, greater compliance (≥80%) with HPONS was largely found in studies (six out of eight) of short intervention periods (< 1-2 weeks), in patients with GI cancers undergoing surgery, and in those trials using HPONS containing omega-3. In studies with poorer compliance (< 80%), the majority of patients were undergoing chemotherapy/radiotherapy across GI, lung or a mix of cancers, receiving HPONS for much longer periods of time. Changes in compliance with HPONS intake over time were illustrated in community patients undergoing chemotherapy, reducing from ≥80% (87.2%−89.5%) compliance in the first 2 weeks of intervention to 40.5%−63.6% in the following 4 weeks (70). Therefore, given the wide variation in intervention durations, the diverse compositions and energy densities of HPONS, with and without omega-3 fatty acids, and the heterogeneity of patient populations and clinical settings, further research is needed to inform more targeted recommendations for clinical practice. Future studies should also explore strategies to overcome common barriers to compliance with ONS among cancer patients, including gastrointestinal intolerance, early satiety, aversions to flavour or texture, and lack of motivation (114, 115).

In addition to compliance, the impact of HPONS on total nutrient intakes is an important consideration, as patients may comply well to the intervention, but the intake of the ONS might suppress patients' appetite and reduce their food intake (thereby not increasing total nutritional intake, which we believe is a fundamental step to improving outcomes, as discussed above). In many trials, including those involving HPONS, data consistently demonstrate that ONS effectively improve overall intake of energy, protein, and various micronutrients, without significantly reducing intake from regular food sources (116). Unfortunately, in this systematic review, reporting of nutritional intake data was heterogeneous and not widely recorded in all the studies assessing clinical outcomes, making synthesis and a comprehensive conclusion difficult. Less than 40% (eleven out of twenty-nine) of studies reported total energy intakes, although of these, ten studies showed higher energy intakes vs. the control group (six of which were statistically significant). Only 28% of studies (eight out of twenty-nine) reported total protein intakes, with significantly greater protein intakes with HPONS vs. control shown in most (six out of eight) studies, an important outcome considering how difficult it can be to meet protein requirements in those with cancer. Furthermore, the systematic review lacked sufficient data to draw conclusions about improvements in the intake of different types and qualities of protein, particularly those rich in key amino acids, from both HPONS and dietary sources, which may be critical for enhancing clinical outcomes. Improving the intake of other nutrients, including omega-3 fatty acids, vitamins, and minerals, may also be important for improving outcomes in patients with cancer. Specifically, omega-3 fatty acids have been shown to play an important role in reducing inflammation, and their use in the nutritional support of patients with cancer has been explored in research over many years (117–121) and featured in guidelines (18, 19, 21) due to their broad range of biological functions. However, among the fourteen studies in this systematic review that included an omega-3 enriched HPONS, although the prescription provided was most often recorded [from 0.92 g to 3.3 g/day, with recommendations in literature ranging from ~600-700 mg (122, 123) to 2 g/day (124, 125)], the total intake of omega-3 fatty acids provided (from HPONS and diet) was not widely explored or linked to specific clinical outcomes. Therefore, further research is needed to explore the optimal composition (dose, timing, and duration) of HPONS, to optimally meet the needs of patients according to cancer type, stage, etc., treatment regimens, surgery, age, and other co-morbidities.

### 4.1 Limitations/more research

There are a number of limitations to this systematic review and meta-analysis. In terms of individual trial methodology, although RoB2 (53) assessments suggested there was a low risk of bias due to outcome measurement, overall, there was low to medium certainty of evidence using GRADE for the outcome of complications (often due to the lack of blinding and use of a placebo which can be more challenging in a nutritional intervention trial). Similarly, the patients included in many trials had mixed nutritional status (with malnourished patients sometimes excluded for ethical reasons), had different cancer locations, types and/or treatments and the prescriptions of HPONS used also varied (dose, timing, composition, and duration) as did the comparators (routine care, dietary advice, hospital diet, etc). This review did not conduct this level of sub-group meta-analyses, which were beyond the scope of the current synthesis. Greater standardisation in research trials will support more robust conclusions.

At a minimum, clear reporting of patients' nutritional status, the composition and actual intake of the intervention, its impact on overall intake, and the inclusion of relevant outcome measures will enhance the quality and interpretability of future systematic reviews. Furthermore, this review only focused on clinical outcomes, whilst many other functional and patient-reported outcomes, such as quality of life, need to be considered, in addition to the economic impact of intervention with HPONS in those with cancer. Although the trials included were undertaken in patients undergoing chemotherapy and/or radiotherapy, the huge range of therapeutic agents and regimens used will also influence findings, and very few trials included the use of immunotherapy, so further research in these groups is particularly recommended.

Finally, this review excluded non-English language studies and grey literature due to resource and feasibility constraints. As a result, there may be a risk of language and publication bias, which may affect the findings. Future reviews should consider including these sources to enhance inclusivity and reduce potential bias whilst ensuring methodological quality and robustness of studies' findings.

## 5 Conclusion

This systematic review and meta-analysis have shown that use of HPONS (including those enriched with omega-3) is associated with a reduction in complications, such as infectious and non-infectious, wound, post-operative and radiotherapy-related complications, in a variety of patients with cancer, across hospital and community settings. Reduced LOS was also observed in patients taking HPONS, often for short periods of time, when undergoing cancer surgery. No effect was observed on hospital readmissions or mortality. The high heterogeneity in patient populations, cancer sites, treatment modalities, and nutritional statuses across included RCTs limits generalisability, with further investigation required. Due to the importance of tackling malnutrition with effective nutritional interventions to improve the outcome of those with cancer, further research is recommended, with robust controlled designs, and greater standardisation of patient groups, cancer treatment regimens and nutritional interventions, to further improve the evidence base to enable recommendations for practise.

## Data Availability

The original contributions presented in the study are included in the article/Supplementary material, further inquiries can be directed to the corresponding author.
