# Supplementary material for: High-protein oral nutritional supplement use in patients with cancer reduces complications and length of hospital stay: a systematic review and meta-analysis
Source: Front Nutr. 2025 Sep 10;12:1654637. doi: 10.3389/fnut.2025.1654637 (PMC12459276; doi:10.3389/fnut.2025.1654637)

| **Appendix**  Supplemental Material 1. List of full references of included publications (N=32, of N=29 studies), with summary of relevant measured outcomes | | |
| --- | --- | --- |
| **Study** | **Publication Title and Journal** | **Measured outcomes** |
| Abouegylah et al. 2024[^66^](#_ENREF_66) | The Role of Oral Nutritional Supplements in Head and Neck Cancer Patients Undergoing Chemoradiotherapy. Healthcare. | Complications |
| Akita et al. 2019[^67^](#_ENREF_67) | The utility of nutritional supportive care with an eicosapentaenoic acid (EPA)-enriched nutrition agent during pre-operative chemoradiotherapy for pancreatic cancer: Prospective randomized control study. Clin Nutr ESPEN. | Energy Intake |
| Aoyama et al. 2022[^68^](#_ENREF_68)  (follow-up study of [^79^](#_ENREF_79)) | Effects of perioperative eicosapentaenoic acid-enriched oral nutritional supplement on the long-term oncological outcomes after total gastrectomy for gastric cancer. Oncol Lett. |  |
| Braga et al. 2002^[69](#_ENREF_69)^ | Preoperative oral arginine and n-3 fatty acid supplementation improves the immunometabolic host response and outcome after colorectal resection for cancer. Surgery. | Compliance  Complications  Length of hospital stay  Readmission to hospital Mortality |
| Dingemans et al. 2023[^70^](#_ENREF_70) | High Protein Oral Nutritional Supplements Enable the Majority of Cancer Patients to Meet Protein Intake Recommendations during Systemic Anti-Cancer Treatment: A Randomised Controlled Parallel-Group Study. Nutrients. | Compliance  Energy and Protein Intake |
| Dou et al. 2020[^71^](#_ENREF_71) | Effect of oral supplements on the nutritional status of nasopharyngeal carcinoma patients undergoing concurrent chemotherapy: A randomized controlled Phase II trial. J Cancer Res Ther. | Compliance |
| Faber et al. 2015[^72^](#_ENREF_72) | Improved body weight and performance status and reduced serum PGE2 levels after nutritional intervention with a specific medical food in newly diagnosed patients with esophageal cancer or adenocarcinoma of the gastro-esophageal junction. J Cachexia Sarcopenia Muscle. | Compliance |
| Faccio et al. 2021^[73](#_ENREF_73)^ | Oral Nutritional Supplementation in Cancer Patients Who Were Receiving Chemo/Chemoradiation Therapy: A Multicenter, Randomized Phase II Study. Nutr Cancer. | Compliance  Energy and Protein Intake |
| Gade et al. 2016[^74^](#_ENREF_74) | The Effect of Preoperative Oral Immunonutrition on Complications and Length of Hospital Stay After Elective Surgery for Pancreatic Cancer--A Randomized Controlled Trial. Nutr Cancer. | Compliance  Complications  Length of hospital stay  Readmission to hospital Mortality |
| Gianotti et al. 2002^[75](#_ENREF_75)^ | A randomized controlled trial of preoperative oral supplementation with a specialized diet in patients with gastrointestinal cancer. Gastroenterology. | Compliance  Complications  Length of hospital stay  Mortality |
| Hanai et al. 2018[^76^](#_ENREF_76) | Prospective randomized investigation implementing immunonutritional therapy using a nutritional supplement with a high blend ratio of ω-3 fatty acids during the perioperative period for head and neck carcinomas. Jpn J Clin Oncol. | Compliance  Complications  Energy Intake |
| Hatao et al. 2017[^77^](#_ENREF_77) | Randomized controlled clinical trial assessing the effects of oral nutritional supplements in postoperative gastric cancer patients. Langenbecks Arch Surg. | Compliance |
| Ibrahim et al. 2021[^78^](#_ENREF_78) | Oral nutritional supplements (ONSs) for cirrhotic patients undergoing liver resection assessed by ultrasound measurement of rectus femoris and anterior tibialis muscles thickness. Randomized clinical trial. Saudi J Anaesth. | Compliance  Complications  Length of hospital stay |
| Ida et al. 2017[^79^](#_ENREF_79) | Randomized clinical trial comparing standard diet with perioperative oral immunonutrition in total gastrectomy for gastric cancer. Br J Surg. | Compliance  Complications  Mortality |
| Kabata et al. 2015^[80](#_ENREF_80)^ | Preoperative nutritional support in cancer patients with no clinical signs of malnutrition--prospective randomized controlled trial. Support Care Cancer. | Complications |
| Kerr et al. 2022[^81^](#_ENREF_81) | Feasibility study of a randomised controlled trial of preoperative and postoperative nutritional supplementation in major lung surgery. BMJ Open. | Compliance  Complications  Length of hospital stay  Readmission to hospital  Mortality |
| Laviano et al. 2020^[82](#_ENREF_82)^ | Safety and Tolerability of Targeted Medical Nutrition for Cachexia in Non-Small-Cell Lung Cancer: A Randomized, Double-Blind, Controlled Pilot Trial. Nutr Cancer. | Compliance  Complications  Mortality |
| Lee et al. 2023[^83^](#_ENREF_83) | Impact of Preoperative Immunonutrition on the Outcomes of Colon Cancer Surgery: Results from a Randomized Controlled Trial. Ann Surg. | Compliance  Complications  Length of hospital stay  Readmission to hospital |
| Lee et al. 2024[^84^](#_ENREF_84) | Oral Protein Supplements Might Improve Nutritional Status and Quality of Life in Elderly Patients after Standard Pancreatic Resection. Nutrients. | Compliance  Energy and Protein Intake |
| Moya et al. 2016[^85^](#_ENREF_85) | Perioperative immunonutrition in normo-nourished patients undergoing laparoscopic colorectal resection. Surg Endosc. | Compliance  Complications  Length of hospital stay  Readmission to hospital  Mortality |
| Pastore et al. 2014^[86](#_ENREF_86)^ | Introduction of an Omega-3 Enriched Oral Supplementation for Cancer Patients Close to the First Chemotherapy: May It Be a Factor for Poor Compliance? Nutrition and Cancer. | Compliance* |
| Ravasco et al. 2005a[^87^](#_ENREF_87) | Impact of nutrition on outcome: a prospective randomized controlled trial in patients with head and neck cancer undergoing radiotherapy. Head Neck. | Energy and Protein Intake |
| Ravasco et al. 2005b[^88^](#_ENREF_88) | Dietary counseling improves patient outcomes: a prospective, randomized, controlled trial in colorectal cancer patients undergoing radiotherapy. J Clin Oncol. | Energy and Protein Intake |
| Ravasco et al.2012[^89^](#_ENREF_89)  (follow-up study of [^88^](#_ENREF_88)) | Individualized nutrition intervention is of major benefit to colorectal cancer patients: long-term follow-up of a randomized controlled trial of nutritional therapy. Am J Clin Nutr. | Complications  Mortality |
| Sanchez-Lara et al. 2014[^90^](#_ENREF_90) | Effects of an oral nutritional supplement containing eicosapentaenoic acid on nutritional and clinical outcomes in patients with advanced non-small cell lung cancer: randomised trial. Clin Nutr. | Compliance  Energy and Protein Intake  Mortality |
| Sathiaraj et al. 2023[^91^](#_ENREF_91) | Effects of a Plant-Based High-Protein Diet on Fatigue in Breast Cancer Patients Undergoing Adjuvant Chemotherapy - a Randomized Controlled Trial. Nutr Cancer. | Energy and Protein Intake |
| Trabal et al. 2010[^92^](#_ENREF_92) | Potential usefulness of an EPA-enriched nutritional supplement on chemotherapy tolerability in cancer patients without overt malnutrition. Nutr Hosp. | Compliance  Complications  Energy Intake |
| Ueno et al. 2022[^93^](#_ENREF_93) | Randomized Phase II Study of Gemcitabine Monotherapy vs. Gemcitabine with an EPA-Enriched Oral Supplement in Advanced Pancreatic Cancer. Nutr Cancer. | Complications  Mortality |
| Van der Meij et al. 2010[^94^](#_ENREF_94) | Oral nutritional supplements containing (n-3) polyunsaturated fatty acids affect the nutritional status of patients with stage III non-small cell lung cancer during multimodality treatment. J Nutr. | Compliance  Energy and Protein Intake |
| Van der Meij et al.2012[^95^](#_ENREF_95)  (follow-up study of [^94^](#_ENREF_94)) | Oral nutritional supplements containing n-3 polyunsaturated fatty acids affect quality of life and functional status in lung cancer patients during multimodality treatment: an RCT. Eur J Clin Nutr. | Complications  Readmission to hospital |
| Yan et al. 2021[^96^](#_ENREF_96) | Perioperative Enteral Nutrition Improves Postoperative Recovery for Patients with Primary Liver Cancer: A Randomized Controlled Clinical Trial. Nutr Cancer. | Length of hospital stay |
| Zietarska et al. 2017^[97](#_ENREF_97)^ | Chemotherapy-Related Toxicity, Nutritional Status and Quality of Life in Precachectic Oncologic Patients with, or without, High Protein Nutritional Support. A Prospective, Randomized Study. Nutrients. | Complications |

*excluded from descriptive summary of compliance data as it reported HPONS discontinuation rates rather than actual intake relative to the prescribed dose

Supplemental Material 2. Meta-analysis of the effects of high-protein ONS on relevant outcomes with risk of bias

**2.1.1 Forest plot for outcome: Complications; HPONS vs. control (N=15)**


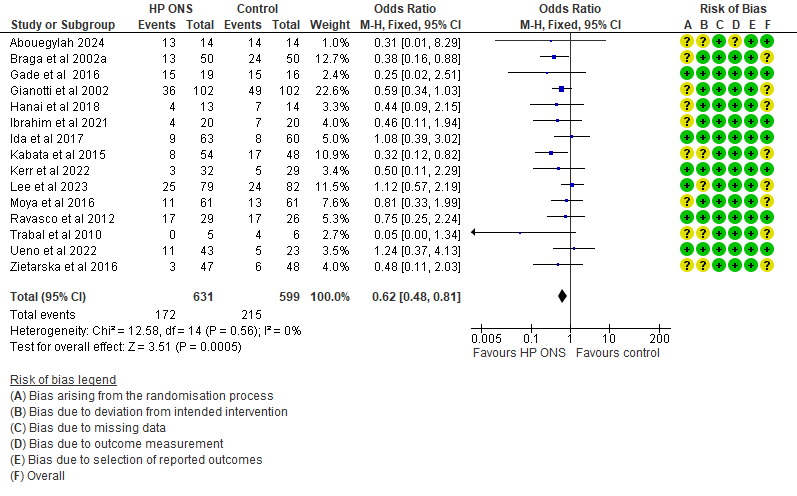


***2.1.2 Sub-group Forest plot for outcome: Complications; HPONS containing omega-3 vs. control (N=9)***


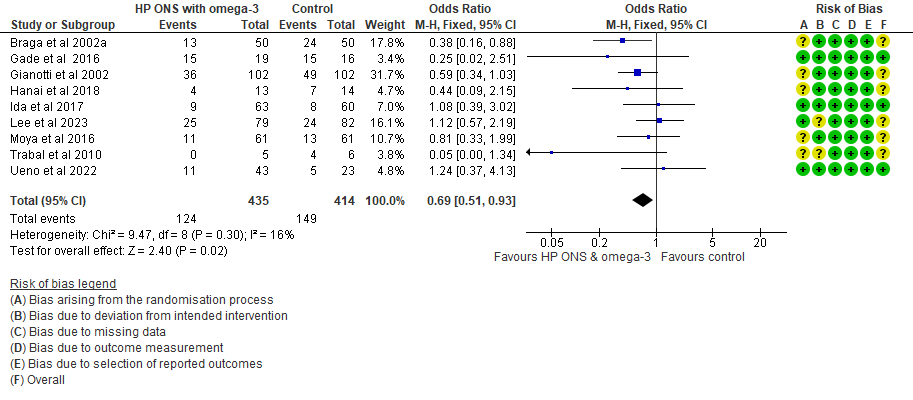


***2.1.3 Sub-group Forest plot for outcome: Complications; HPONS without omega-3 vs. control (N=6)***


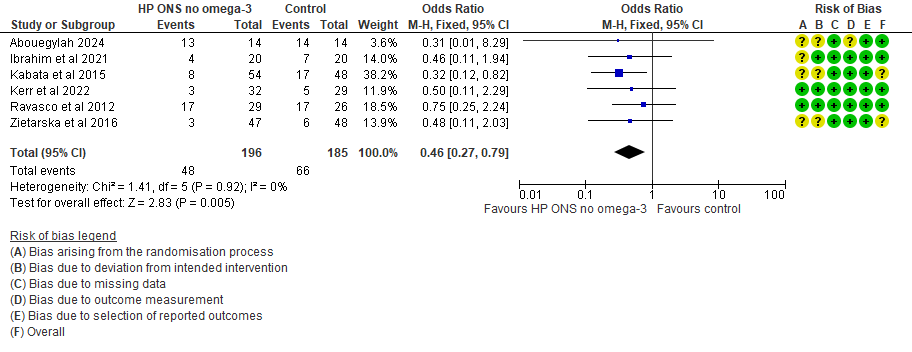


***2.2.1 Forest plot for outcome: Length of hospital stay; HPONS vs. control (N=8)***


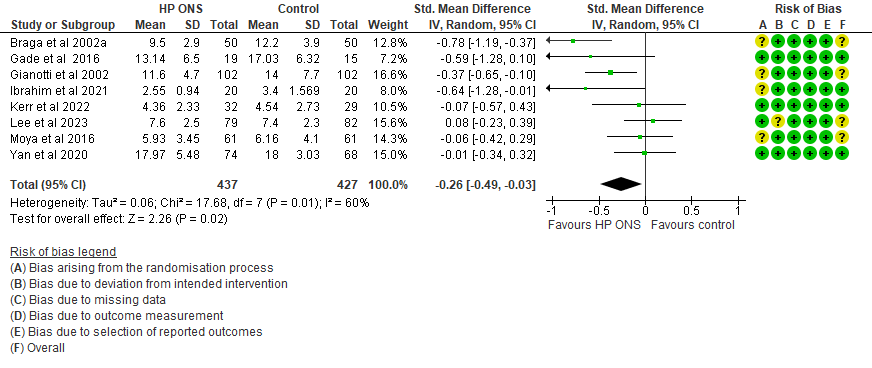


**2.2.2 Forest plot for sensitivity analysis for outcome: Length of hospital stay; HPONS vs. control**

**
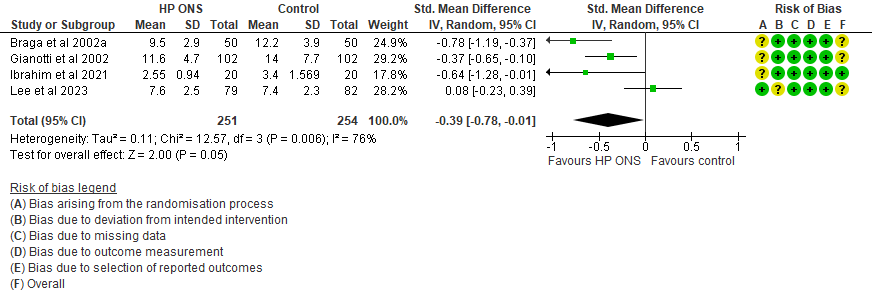
**

**2.3.1 Forest plot for outcome: Hospital readmissions; HPONS vs. control (N=5)**


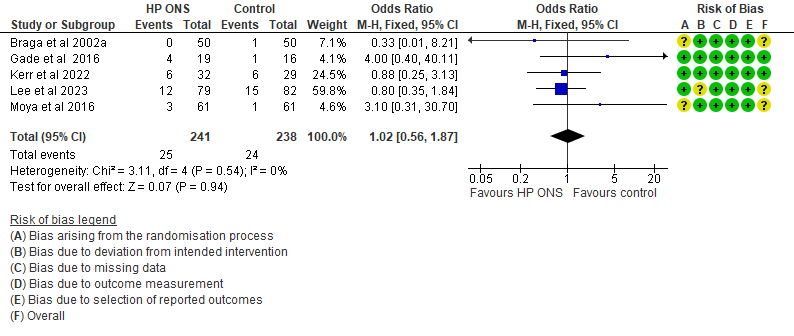


**2.4.1 Forest plot for outcome: Mortality; HPONS vs. Control (N=7)**


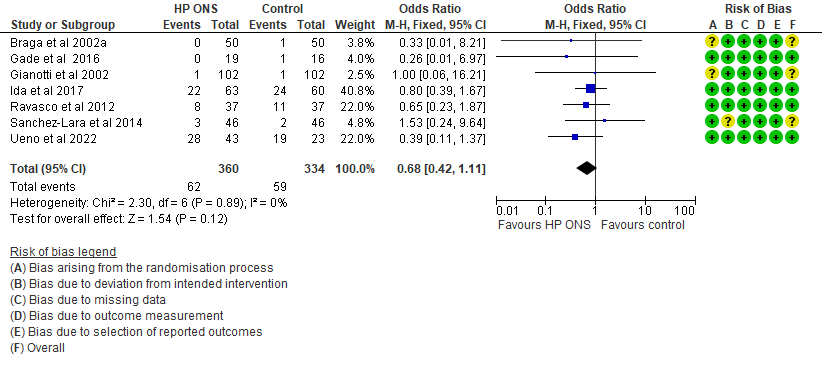

Supplement: Supplementary file 1 [file Table_1.docx]
